# Kansui-liquorice enhances the “water-expelling” effect of Gansui Banxia decoction in rats with malignant ascites by targeting the NPs/NPRs/cGMP/PKGⅡ pathway and T cell immunity

**DOI:** 10.3389/fphar.2025.1557717

**Published:** 2025-04-28

**Authors:** Min Huo, Haiyan Liu, Shaohong Chen, Lingling Xiu, Xue Yu, Gansheng Zhong

**Affiliations:** School of Traditional Chinese Medicine, Beijing University of Chinese Medicine, Beijing, China

**Keywords:** kansui-liquorice, Gansui Banxia decoction, network pharmacology, pharmacokinetics, water-expelling, T Cell immunity, natriuretic peptide system

## Abstract

**Ethnopharmacological relevance:**

The combination of *Euphorbia kansui* Liou ex S.B.Ho (kansui) and *Glycyrrhiza uralensis* Fisch (liquorice) is contraindicated in Chinese medicine, but whether it can be used in clinical practice remains controversial. The classic formula, Gansui Banxia decoction (GBD), contains kansui and liquorice, which is effective in treating an abnormal accumulation of body fluids, such as malignant ascites (MA); however, the contraindications of kansui and liquorice have limited its clinical application.

**Aim of the study:**

This study aims to provide a theoretical basis for the rational application of kansui-liquorice by investigating its role and mechanism in GBD.

**Materials and methods:**

LC-MS/MS was used to detect the metabolic differences of - glycyrrhetinic acid, glycyrrhizic acid, glycyrrhizin, glycyrrhizin, glycyrrhizin terpinolipid A, and paeoniflorin - in the liquid of MA rats before and after taking GBD. Network pharmacology was employed to predict the potential targets and mechanisms of GBD in the treatment of MA. The experimental validation was still using MA rats as a model. Flow cytometry was used to assess the expression of immune cells in blood and ascites, and the proliferation and development of T cells in bone marrow and thymus. Elisa was used to detect the content of atrial natriuretic peptide (ANP) and brain natriuretic peptide (BNP) in blood. Western blot and qRT-PCR were used to detect the expression of NPs/NPR-A/cGMP/PKG II pathway-related gene and proteins in kidney. The MA model was established by intraperitoneal injection of walker-256 cells at a concentration of 2 × 106/mL and an injection volume of 1 mL. The model was successfully established when the abdominal cavity was obviously distend and touched with a water-shaking sound, and ascites could be seen after opening the abdominal cavity.

**Results:**

We confirmed that GBD containing kansui-liquorice could promote the metabolism of liquorice and reduce the precipitation of toxic substances (kansuinine A). It may also target cellular immunity to exert a drug effect. Further experimental verification found that GBD containing kansui-liquorice could promote the activation of the NPs/NPRs/cGMP/PKGⅡ pathway and exert a diuretic effect in MA rats. Besides that, it could increase the proportion of CD8CD28 T cells, reduce the proportion of immune-suppressing cells, and maintain the stability of the developmental environment of the T cells.

**Conclusion:**

We believe that kansui and liquorice are important components of GBD, and their combination could promote GBD to promote the clinical remission of MA through direct (activation of the NPs/NPRs/cGMP/PKGⅡ pathway) and indirect (regulating T-cell immunity) water-expelling effects.

## Graphical Abstract

**Figure:**
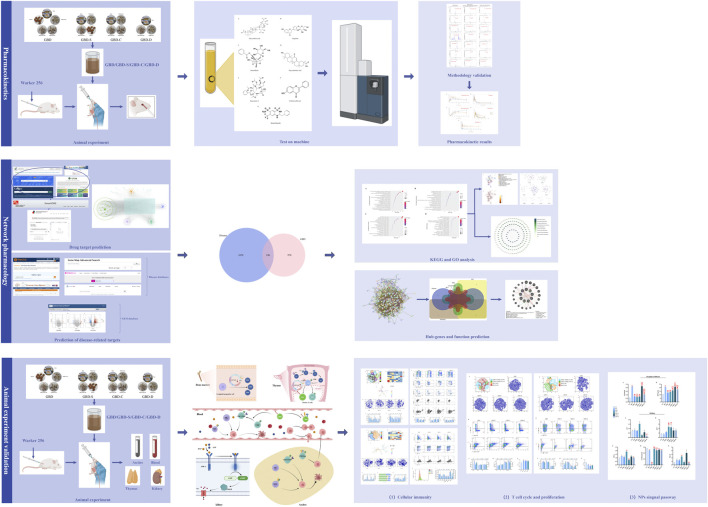
Through pharmacokinetics, network pharmacology, and experimental validation, we believe GBD with kansui-liquorice may through direct (activation of the NPs/NPRs/cGMP/PKGⅡ pathway) and indirect (regulating T-cell immunity) water-expelling effect to promote the clinical remission of MA rats. Created with BioRender.com.

## Mechanism Graph

**Figure:**
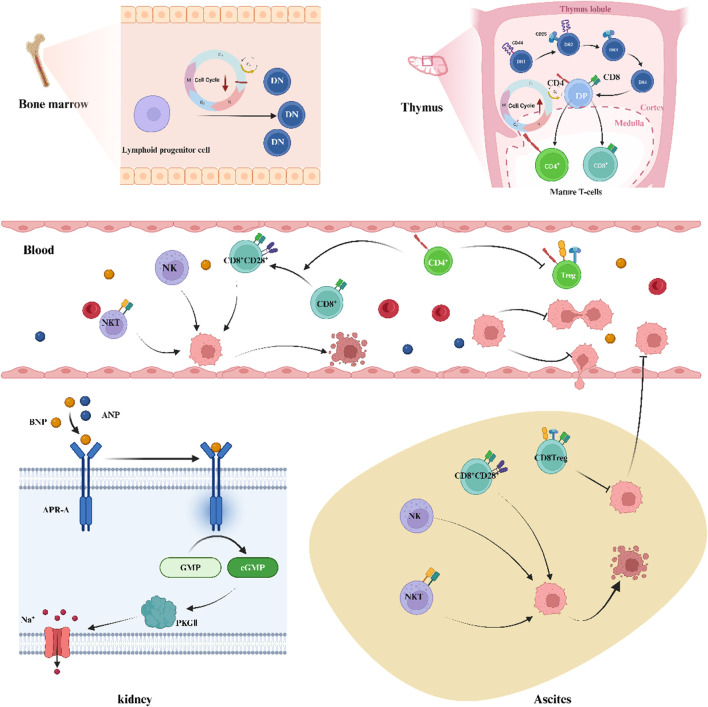
This graph demonstrates the possible mechanism of kansui-liquorice enhances the efficacy of GBD. kansui-liquorice in GBD could: (1) enhance the activation of the NPs signalling pathway, which plays a direct water-expelling (diuretic) effect; (2) increase the proportion of CD8CD28 T cells in the PB and TME, and decrease the proportion of immune-suppressing cells in the PB (Treg cells) and TME (CD8 Treg cells), and promote the development of T cells to enhance the cellular immune, which exerts an indirect water-expelling effect. Created with BioRender.com.

## 1 Introduction

Traditional Chinese medicine (TCM) is indispensable in the Chinese medical system due to its unique theoretical system. In TCM, some forbidden/avoidable combinations of Chinese medicines are called “eighteen antagonisms” and “nineteen incompatibilities.” For example, *Veratrum nigrum* L (lilu) and *Panax ginseng* C.A. Mey (ginseng). *Euphorbia kansui* Liou ex S.B.Ho (kansui) and *Glycyrrhiza uralensis* Fisch (liquorice) are categorized as the “eighteen antagonisms,” which are prohibited combinations; however, this view has been questioned, and there is no consensus on whether these ingredients could be used together. According to a part of TCM theory, kansui-liquorice in combination with other drugs can exert a water-expelling effect without any obvious toxic side effects.

Gansui banxia decoction (GBD) is recorded as a classic prescription in TCM, and is composed of kansui and liquorice. Due to its powerful water-draining effect, GBD has been used to treat diseases with abnormal fluid accumulation, such as cancer ascites, cirrhosis ascites, and renal edema (Liu et al., 2014). However, kansui-liquorice incompatibility limits its clinical application. GBD expels water by enhancing diuresis and anti-tumor immunity in rats with malignant ascites (MA) (Huo et al., 2024); however, the effect of kansui-liquorice combination on GBD has not been clarified.

Therefore, this study aims to optimize the composition of GBD by investigating the role and mechanism of kansui-liquorice in GBD, and to provide a theoretical basis for the clinical rational application of GBD in treating MA.

## 2 Material and methods

### 2.1 Pharmacokinetics of GBD with or without kansui-liquorice in MA rats

A rapid liquid chromatography-tandem mass spectrometry (LC-MS/MS) method was developed to determine the amounts of glycyrrhizic acid, liquiritin, paeoniflorin, glycyrrhetinic acid, and kansuinine A after intragastric administration of GBD with or without kansui or/and liquorice.

#### 2.1.1 Drug preparation

GBD is composed of five TCM plants, including *E. kansui* Liou ex S.B.Ho (kansui, GS), *G. uralensis* Fisch (liquorice, GC), *Paeonia lactiflora* Pall (radix paeoniae alba, BS), *Pinellia ternata* (Thunb.) Makino (pinellia ternata, BX), and *Apis cerana* Fabricius (honey, FM). The relevant information of each plant is shown in Table 1. The plant’s name had been checked with http://www.worldfloraonline.org (Last accessed on 2024-9-21). The active ingredients of GBD had been identified in the early stage of the study (Huo et al., 2024).

**TABLE 1:** The composition of GBD.

Herbs	Chinese name	Latin name	Processing method^a^	Drug dose (g/kg rats/d)^b^
Kansui	甘遂 (Gansui, GS)	*Euphorbia kansui* Liou ex S.B.Ho	Crude (omit the vinegar process)	0.11
Liquorice	甘草 (Gancao, GC)	*Glycyrrhiza uralensis* Fisch	Stir-frying with honey	1.67
Radix paeoniae alba	白芍 (Baishao, BS)	*Paeonia lactiflora* Pall	—	1.5
Pinellia ternata	半夏 (Banxia, BX)	*Pinellia ternata* (Thunb.) Makino.	Made with liquorice and quicklime	0.9
Honey	蜂蜜 (Fengmi, FM)	*Apis cerana* Fabricius	—	1.5

^a^ The specific processing methods are based on Pharmacopoeia of the People’s Republic of China: Part I.

^b^ The conversion factor is about 0.02 based on the body surface area of a 200 g rat and 60 kg human, which means that for every 1 g of Chinese medicine taken by an adult, a 1-kg rat takes 0.1 g.

GBD has the best efficacy and no apparent toxicity when kansui is 0.11 g and liquorice is 1.37 g (conversion ratio is calculated based on body surface area of a 200 g rat and 60 kg human using a conversion factor of 6.2 for rats to humans) (Liu, 2014). Based on this previous experiment, we explored the role and pharmacodynamic mechanism of kansui-liquorice in GBD. Thus, in this experiment, the dosage of GBD was as follows: 1.5 g of radix paeoniae alba, 0.9 g of pinellia ternata, 1.5 g of honey, 1.67 g of liquorice, and 0.11 g of kansui. Kansui, liquorice, and both plants were removed from the decoction to form GBD-S, GBD-C, and GBD-D, respectively. All drug solution concentrations, administered doses and rats grouping are shown in Table 2.

**TABLE 2:** The composition of experimental drugs.

Drugs	Composition	Concentration	Dosage	Groups
GBD	Radix paeoniae alba; Pinellia ternata; Honey; Liquorice; Kansui	0.568 g/mL	5.68 g/kg/d	GBT
Gansui Banxia decoction remove kansui (GBD-S)	Radix paeoniae alba; Pinellia ternata; Honey; Liquorice	0.557 g/mL	5.57 g/kg/d	GBT-S
Gansui Banxia decoction remove liquorice (GBD-C)	Radix paeoniae alba; Pinellia ternata; Honey; Kansui	0.401 g/mL	4.01 g/kg/d	GBT-C
Gansui Banxia decoction remove kansui and liquorice (GBD-D)	Radix paeoniae alba; Pinellia ternata; Honey	0.39 g/mL	3.9 g/kg/d	GBT-D

For the preparation of GBD, the radix paeoniae alba, pinellia ternata, and liquorice were soaked for 30 min. The medicines were decocted twice, the liquid was mixed, the honey was added, and the liquid was concentrated to 0.557 g/mL by the water bath heating method. Finally, 0.011 g of kansui was added to each milliliter of liquid for further use. The other TCM decoctions were also prepared as above.

The radix paeoniae alba (no. 120200602), pinellia ternata (no. 009200702), liquorice (no. 099200902), and kansui (no. 011181002) were purchased from Anguo Jinglongkang Pharmaceutical Co., LTD., and processed and sliced into the necessary pieces for the experiment. These were qualified as appropriate herbal pieces by a senior laboratory technician.

#### 2.1.2 Animals

Wistar rats (male, 200 ± 20 g; age, 6 weeks) were supplied by Charles River Laboratories, Beijing, China [Certificate of Conformity: SYXK (Beijing) 2020-0033], and acclimated for 1 week prior to use in the experiments. The rats were raised in a standardized environment with controlled conditions (temperature, 22°C ± 2°C; humidity, 50∼70%; light/dark cycle, 12 h). Food and water were freely available. All animal experiments were approved by the Medical and Laboratory Animal Ethics Committee of Beijing University of Chinese Medicine (No. BUCM-2020031502-1028).

Female rats are more affected by hormonal cyclicity, and since this study contains hormone assays, all male rats were used in this experiment instead of half males and half females.

#### 2.1.3 Model preparation and treatment

The experimental rats were fed adaptively for 7 days and then randomly divided into a GBD group (GBT), GBD-S group (GBT-S), GBD-C group (GBT-C), and GBD-D group (GBT-D). Each group consisted of 6 rats for a total of 24 rats.

Walker-256 cells were injected into the abdominal cavity of the experimental rats (except the CG) at a concentration of 2 × 10^6^/mL. The ascites model was successfully established when the abdominal cavity was obviously distended, the abdominal cavity was touched with a water-shaking sound, and ascites was seen after opening the abdominal cavity. The rats with successful modeling were taken for subsequent experiments.

GBT groups is rats given GBD solution, GBT-S group is rats given GBT-S solution, GBT-C group is rats given GBT-C solution, and GBT-D group is rats given GBD-D solution.

The corresponding drugs were given to the experimental rats after modeling 3 days. The rats in the CG and the MG were given 10 mL/kg deionised water; the other drug groups were given the corresponding drugs as shown in Table 2.

#### 2.1.4 Collection of plasma samples

200 μL of blood was extracted from the orbital venous plexus before and after intragastric administration at 10, 20, 40, 60, 120, 240, 360, 480, 720, and 1,440 min, and then centrifugated to obtain plasma.

#### 2.1.5 Preparation of reference solution and internal standard solution

##### 2.1.5.1 Reference solution preparation

We weighed 10.0 mg of liquiritin reference standard (Chengdu Pufei De Biotech Co., LTD, Lot number: 20,080,809), added methanol (Shanghai Macklin Biochemical Technology Co., Ltd., Lot number: 126,273) to make up 10 mL, to prepare a stock solution of 1 mg/mL. We then accurately weighed 10.0 mg of glycyrrhizic acid (Chengdu Pufei De Biotech Co., LTD, Lot number: 20,070,203), glycyrrhetinic acid (Chengdu Pufei De Biotech Co., LTD, Lot number: 20,041,002), kansuinine A (Chengdu Pufei De Biotech Co., LTD, Lot number: 20,060,807), and paeoniflorin reference standards (Chengdu Pufei De Biotech Co., LTD, Lot number: 20,080,816), added methanol to make up to 5 mL, to prepare a stock solution of 2 mg/mL. The stock solutions were stored in a refrigerator at 4°C for later use.

##### 2.1.5.2 Internal standard solution preparation

We weighed 10.0 mg of bendrofluazide (China Institute for Food and Drug Control, Lot number: 100007-200703) and 6-hydroxyflavone (6-HF) (China Institute for Food and Drug Control, Lot number: 111787-201002), and added methanol to make up to 10 mL, to prepare a stock solution of 1 mg/mL. The stock solution was used to prepare internal standard solutions with concentrations of 50 ng/mL for bendrofluazide, and 100 ng/mL for 6-HF.

#### 2.1.6 Plasma sample processing method

We removed 20 μL of plasma from the experimental rats (blank plasma), and added 20 μL of reference solution, 10 μL of internal standard solution, and 50 μL of 0.01% acetic acid methanol. The mixture was vortexed for 1 min, centrifuged at 14,000 rpm for 15 min at 4°C, and the supernatant fluid was analysed.

#### 2.1.7 Measurement conditions

##### 2.1.7.1 Chromatographic conditions

Column: Waters CORTECS C18 column (4.6 × 50 mm, 2.7 µm, Waters, Milford MA, United States). Mobile phase: A phase was 0.05% formic acid water; B phase was acetonitrile (Shanghai Jizhi Biochemical Technology Co., Ltd., Lot number: C12617706). For the elution gradient, see Supplementary Table 1. Flow rate: 0.6 mL/min. Column temperature: 25°C. Injection volume: 5 μL. Needle wash solution: methanol, water, and acetone (Shanghai Macklin Biochemical Technology Co., Ltd., Lot number: 127,363) (methanol: water: acetone = 3: 3: 2). Automatic sampler temperature control: 10°C. Valve switching: From 1.8 to 4.3 min, switch to mass spectrometry detection, waste flow for the remaining time.

##### 2.1.7.2 Mass spectrometry conditions

Using an electrospray ionization (ESI) source, the needle pump method was first employed to select and optimize the parameters of the components to be tested, such as fragment ions, collision energy, and declustering potential. Negative ion scanning was conducted in Multi Reaction Monitoring (MRM) mode, and the main mass spectrometry parameters are shown in Supplementary Table 2. Fragment information for each compound can be found in Supplementary Table 3). To maximize the detection sensitivity, entrance potential, collision cell exit potential, ion source gas 1, ion source gas 2, and the position of the spray needle were optimized separately.

#### 2.1.8 Methodological investigation

##### 2.1.8.1 Specificity investigation

We took 20 μL of blank plasma A (was not added the reference and internal standard solutions), blank plasma B (was added the reference and internal standard solutions) and plasma C (were taken from rats after intragastric administration and added the reference and internal standard solutions), and processed the sample according to Section 2.1.6.

We examined five compounds, including glycyrrhizic acid, liquiritin, glycyrrhetinic acid, paeoniflorin, and kansuinine A. For paeoniflorin, glycyrrhizic acid and liquiritin, we chose 6-HF as the internal standard; for glycyrrhetinic acid and kansuinine A, bendrofluazide was chosen. The structural diagram of the above chemical composition is shown in Supplementary Figure 1.

The MRM results of each component are shown in Supplementary Figure 2. The retention times of paeoniflorin, liquiritin, glycyrrhizic acid, glycyrrhetinic acid and kansuinine A are1.87 min, 1.94 min, 2.39 min, 3.88 min, and 3.40 min, respectively, whereas the retention times of the internal standards of bendrofluazide and 6-HF are 2.75 min and 2.70 min, respectively. On recording the chromatogram, the results show that endogenous substances in the plasma did not interfere with the determination (Supplementary Figure 2).

##### 2.1.8.2 Extraction recovery and matrix effect

Blank plasma, treated according to Section 2.1.6 was named as pre-extraction spiked plasma samples (A); standard solutions were added after precipitation named post-extraction spiked plasma samples (B). The samples (C) were obtained when blank plasma was changed to ultrapure water according to the method used to extract sample B.

The recoveries of the five chemical compositions from the rat plasma at three levels (low, medium, and high) were determined by comparing the peak areas of spiked plasma samples before and after extraction (A/B). This ratio gives the percentage recovery. The matrix effect (B/C) was measured by comparing the pear-area ratios of the analytes in the post-extraction spiked plasma (B) with those of the same amount of standard solutions (sample C) in the mobile phase. The recovery and matrix effects of the five analytes at three QC levels (low, medium, and high) were repeated for three replicates.

The results of the extraction recovery and matrix effects of the five chemical compositions are shown in Supplementary Table 4, showing that the method can meet the requirements of the biological sample analysis of extraction rate and the matrix effect.

##### 2.1.8.3 Standard curve and quantitative range

We took 20 μL plasma from the CG rats, and added 20 μL of a series of concentrations of reference solution and 10 μL of internal standard solution to prepare plasma samples containing 0.05, 0.1, 0.2, 0.5, 1, 2.5, 5, 12.5, 25 ng/mL of liquiritin and kansuinine A; 0.5, 1, 2, 5, 10, 25, 50, 125, 250 ng/mL of glycyrrhizic acid and glycyrrhetinic acid; and 1.5, 3, 6, 15, 30, 75, 150, 375, 750 ng/mL of paeoniflorin, and then processed and analysed according to Section 2.1.6.

The peak area ratio (y) of the analyte to internal standard in plasma samples was used as the ordinate, and the concentration ratio (x) of the analyte to internal standard as the abscissa. We performed regression calculations using the weighted least squares method, with weight coefficient of 1/X^2^, to obtain the regression equation and correlation coefficient of each compound. The results are shown in Supplementary Table 5, which indicates that the five chemical compositions have a good linear relationship and high sensitivity.

##### 2.1.8.4 Precision and accuracy

We then took 20 μL of blank plasma mixed with reference solution to prepare low, medium, and high concentrations (with liquiritin and kansuinine A concentrations of 0.15, 1, and 20 ng/mL; glycyrrhetinic acid and glycyrrhizic acid concentrations of 1.5, 10, and 200 ng/mL; and paeoniflorin concentrations of 4.5, 30, and 600 ng/mL) of plasma samples in triplicate. Following the procedures under Section 2.1.6, we performed a simultaneous analysis with a standard curve. We then calculated the intra-day precision and accuracy, as shown in Supplementary Table 6, which showed that the accuracy and precision of the method met the requirements of the analysis of biological samples.

##### 2.1.8.5 Stability evaluation

We took 20 μL of blank plasma mixed with reference solution to prepare low, medium, and high concentrations of plasma samples, as per Section 2.1.8.4. Their stability was investigated after being left at room temperature for 2 h, three repeated freeze-thaw cycles at −40°C, and placed in the autosampler for 6 h before processing the plasma samples. The stability evaluation results are shown in Supplementary Table 7, which indicates that the five chemical compositions remained stable during the experiment.

### 2.2 Network pharmacology

#### 2.2.1 Screening of active compounds of GBD

Chemical compounds of GS, GC, BX, BS and FM were collected from the Traditional Chinese Medicine Systems Pharmacology Database (TCMSP: https://old.tcmsp-e.com/tcmsp.php, version 2.3, updated on May 2014, accessed April 2023) (Ru et al., 2014), the Encyclopedia of Traditional Chinese Medicine (ETCM: http://www.tcmip.cn/ETCM/, version 2.0, updated on June 2022, accessed April 2023) (Xu et al., 2019), and the Database of Traditional Chinese Medicine and Chemistry (https://organchem.csdb.cn/scdb/main/tcm_introduce.asp, accessed April 2023) (Shanghai Institute of Organic Chemistry Chinese Academy of Sciences, 2014). The above databases are systems pharmacology platforms of Chinese herbal medicines, which summarise information such as the chemical compounds therein. In addition, the other components were obtained by a literature search through China National Knowledge Infrastructure (CNKI: https://www.cnki.net/, accessed April 2023) and PubMed (https://pubmed.ncbi.nlm.nih.gov/, accessed April 2023). Through these databases, we searched and summarised the chemical components of GBD that have been discovered.

The bioactive compounds were screened using Swiss ADME (http://www.swissadme.ch/, released 2017, accessed May 2023) (Daina et al., 2017). The screening criteria were as follows: (1) the “GI absorption” was set as “High,” as the condition that the drug could be absorbed, which was used to screen the bioactive compounds with better oral bioavailability; (2) the compounds with two or more “Yes” results out of “Druglikeness” (Lipinski, Ghose, Veber, Egan, Muegge) were selected as bioactive compounds.

#### 2.2.2 Candidate therapeutic targets of GBD

The therapeutic targets of active compounds in GBD were predicted using Swiss Target Prediction (http://swisstargetprediction.ch/, updated on May 2019, accessed May 2023) (Daina et al., 2019), and the targets with probability >10% were screened.

#### 2.2.3 MA-related targets

We searched for MA-related targets using the following keywords: “malignant ascites,” “malignant hydrops,” and “malignant effusion” at GeneCard (https://www.genecards.org/, version 5.12, updated on September 2022, accessed December 2022) (Safran et al., 2021; Stelzer et al., 2016), OMIM (https://www.omim.org/, updated on September 2022, accessed December 2022) (McKusick-Nathans Institute of Genetic Medicine, 1966), Drugbank (https://go.drugbank.com/, version 5.1.9, updated on January 2022, accessed December 2022) (Knox et al., 2024), the Therapeutic Target Database (TTD: https://db.idrblab.net/ttd/, version 4.3.02, release on August 2011, accessed December 2022) (Zhou et al., 2024), and PharmGKB (https://www.pharmgkb.org/, released 2001, accessed December 2022) (Whirl-Carrillo et al., 2012; Whirl-Carrillo et al., 2021). These databases summarise the pathogenic targets of diseases. A three-fold median was used as the screening condition for key targets in the GeneCard database.

#### 2.2.4 Differentially expressed genes of MA

“Malignant ascites,” “malignant hydrops,” and “malignant effusion” were used as keywords to search the MA-related datasets in the Gene Expression Omnibus datasets (GEO: https://www.ncbi.nlm.nih.gov/geo/, accessed September 2023) (Barrett et al., 2013), and the species was selected as “*Homo sapiens*.” Based on the relevance of the study, GSE73168 (Gao et al., 2019), GSE39204 (Abiko et al., 2013; Ukita et al., 2022), and GSE15831 (Bissels et al., 2009) were finally selected for DGE analysis. SVA and limma packages in R were used for multi-chip joint analysis, with batch correction. The thresholds for the identification of DEGs were |logFC|>1 and *P* < 0.05. Because the three datasets use different detection platforms, the DGEs were analysed separately and then merged.

#### 2.2.5 Acquisition of key therapeutic targets of GBD

The MA-related targets screened in Section 2.2.3 and the DEGs of MA screened in Section 2.2.4 were merged, and then intersected with the therapeutic targets of GBD screened in Section 2.2.2 using the Venn online analysis tool (https://bioinformatics.psb.ugent.be/webtools/Venn/). The acquired intersection is the key therapeutic target of GBD in treating of MA.

#### 2.2.6 Gene ontology and kyoto encyclopedia of genes and genomes enrichment analysis

The intersection genes obtained from Section 2.2.5 were analysed for GO and KEGG enrichment using the Metascape (https://metascape.org, Metascape 3.5, updated on June 2023, accessed October 2023) (Zhou et al., 2019) online analysis tool. GO functional enrichment analysis includes GO Biological Processes (BP), Cellular Components (CC), and Molecular Functions (MF). The results were visualized using the “ggplot2” package in the R language. In addition, the key functional modules under GO BP in the results were analysed to explore the characteristics of intersection genes on cell BP. Finally, the KEGG database (https://www.genome.jp/kegg/, version 108.1, updated on November 2023, accessed November 2023) was used to find interrelationships between pathways in the KEGG analysis results, and the correlations were imported into Cytoscape 3.9.1 for visualization.

#### 2.2.7 Topological and cluster analyses of the protein-protein interaction network

The key therapeutic targets of GBD obtained from Section 2.2.5 were imported into the STRING online database (https://cn.string-db.org/, accessed October 2023) (Szklarczyk et al., 2023) to analyse PPI, and the default parameters were maintained. Then, the generated PPI network TSV file was loaded into Cytoscape 3.9.1 software to draw the PPI network diagram.

#### 2.2.8 Selection and analysis of hub genes

The hub genes were identified using the cytoHubba plug-in of Cytoscape. We used six common algorithms (Degree, MCC, MNC, EPC, Radiality, and Closeness) to evaluate and select the hub genes. Subsequently, we constructed a co-expression network of these hub genes via GeneMANIA (http://www.genemania.org/, accessed November 2023) (Warde-Farley et al., 2010), which is a reliable tool to identify internal associations in gene sets. GeneMANIA is an exceptionally large functional association database that finds other genes related to a set of input genes. Given a list of query genes, GeneMANIA finds functionally similar genes using extensive genomic and proteomic data. In this model, it weights each functional genomic dataset based on the predicted value of the query. Another use of GeneMANIA is gene function prediction. Given a query list of hub genes, GeneMANIA extends the list with functionally similar genes that it identifies using available genomics and proteomics data. Thus, through GeneMANIA, we predicted the functions of the hub genes.

### 2.3 Experimental verification

#### 2.3.1 Drug preparation

The drug preparations were the same as in Section 2.1.1. To explore the diuretic effect of GBD, we chose furosemide as the positive drug. The dosage of furosemide was chosen to be the high value of the initial dose (40 mg/d for adults) according to the instructions, to prevent excessive diuresis and adverse reactions. Furosemide (no. 2003037) was purchased from Tianjin Lisheng Pharmaceutical Co., LTD (Tianjin, China).

#### 2.3.2 Animals

The sex, age, feeding method, and other relevant information of experimental animals were the same as Section 2.1.2.

#### 2.3.3 Model preparation and treatment

The experimental animals’ groupings and the model preparations were similar to those in Section 2.1.3, but we added control group (CG), model group (MG), and positive medicine group (PMG). Each group consisted of 10 rats for a total of 70 rats. The criteria for successful modeling of ascites model are shown in Section 2.1.3. CG is rats without any treatment, MG is rats only injected intraperitoneally with Walker-256 cells without giving any other treatment, PMG is rats given positive medicine.

Then, the corresponding drugs were administered to the experimental rats on the first day after modeling, which was similar to Section 2.1.3, and we chose furosemide as the positive medicine. The rats in the PMG (concentration, 0.4 mg/mL) were administered 4 mg/kg/d. The body condition of MA model rats deteriorates sharply 7 days after injection of cancer cells (Huo et al., 2024). To prevent the rats from being in a cachexia state, the experimental lasted for 7 days.

#### 2.3.4 Sample collection

Ascites: 10 mL of ascites was extracted and placed on ice for later use. Plasma: rats were intraperitoneally injected with 2% pentobarbital (Sigma, no. 20170318). After anesthesia, the abdominal aortic blood was collected and divided into two parts, and placed in anticoagulant tubes, named Blood A and Blood B. Blood A was left at 4°C for more than 30 min, and centrifuged at 3,500 rpm for 15 min. The plasma was collected for related detection. Blood B was placed at 4°C for later use. Kidneys: Bilateral kidneys of rats were collected, and one side was stored at −80°C (Kidney A) to detect cGMP protein content, and the other side was placed at −20 °C (Kidney B) to examine the expression of genes involved in the NPs pathway. Thymus and bone marrow: Single cell suspensions were prepared as described previously (Huo et al., 2024).

#### 2.3.5 Enzyme-linked immunosorbent assay

The protein content of atrial natriuretic peptide (ANP) (Beijing Huaying Biotechnology Research Institute, no. 20211125), brain natriuretic peptide (BNP) (Beijing Huaying Biotechnology Research Institute, no. 20211125) in plasma, and cGMP (Cayman, no. 581021) in Kidney A were analysed using rat ELISA kits by the manufacturer’s instructions.

#### 2.3.6 qRT-PCR

In this experiment, the gene expressions of ANP, BNP, NPR-A, and PKGⅡ in the kidneys were determined by RT-PCR. Total RNA extraction: Total RNA was extracted from Kidney A according to the procedures specified in the Tissue RNA Small Amount Extraction Kit (Shanghai Meiji Biomedical Technology Co., LTD., no. 00073877), and stored at −80°C. RNA purity determination: The concentration of total RNA was read and recorded by an ultramicro spectrophotometer. The purity of RNA was analysed according to the OD values at 260 nm/280 nm, and the samples with OD values between 1.8 and 2.0 were selected. Reverse transcription: The reverse transcription system was prepared according to Supplementary Table 8. The reaction conditions for reverse transcription were as follows: 42°C for 60 min, 70°C for 5 min, and 12°C for ∞. After reverse transcription, the cDNA was diluted five times with sterile enzyme-free water (DEPC) and stored at −20°C. Amplification: The amplification systems were prepared according to Supplementary Table 9, and the conditions for the amplification reaction were 95°C for 15 min, 95°C for 10 s, and 60°C for 30 s for a total of 40 cycles. The results were analysed by 2^−ΔΔCt^; β-actin was used as the internal reference; and the primer sequences are shown in Supplementary Table 10.

#### 2.3.7 Flow cytometry

The extraction of immune cells from Blood B and ascites and their subsequent marking with antibodies were conducted according to previous experiments (Huo et al., 2024). The fluorescent colors of antibodies are shown in Supplementary Table 11.

#### 2.3.8 Cell cycle

The single cells of the thymus and bone marrow (abbreviated as marrow) were marked with CD3, CD4, CD8, FVS 780, Ki-67, and 7AAD antibodies as per previous experiments (Huo et al., 2024). The fluorescent dyes and producers of each antibody are shown in Supplementary Table 12.

The Ki-67 antibody and 7-AAD markers within the cell were used to distinguish between different cycles. Ki-67 was significantly expressed in all but the GO phase. 7-AAD belongs to the DNA dye, which can distinguish all cell cycles except GO and G1 phases. Therefore, we could distinguish G0, G1, S, and G2-M phases by Ki-67 and 7-AAD double staining.

### 2.4 Statistical analysis

All results are displayed as means ± standard error. The differences among groups were tested by one-way analysis using SPSS software version 22. Results were considered to be statistically significant when *P* < 0.05.

## 3 Results

### 3.1 Pharmacokinetics of GBD with or without kansui-liquorice in MA rats

In this experiment, we detected five components in the plasma of MA rats, including liquiritin, glycyrrhetinic acid, glycyrrhizic acid, paeoniflorin, and kansuinine A, and then compared the pharmacokinetic differences of these components in GBD with or without kansui-liquorice. The assay (Section 2.1.8) was proven to be sensitive enough to determine these analytes in rat plasma, and the developed and validated method was applied to the pharmacokinetic evaluation of the five components after intragastric administration of the experimental rats.

**FIGURE 1:**
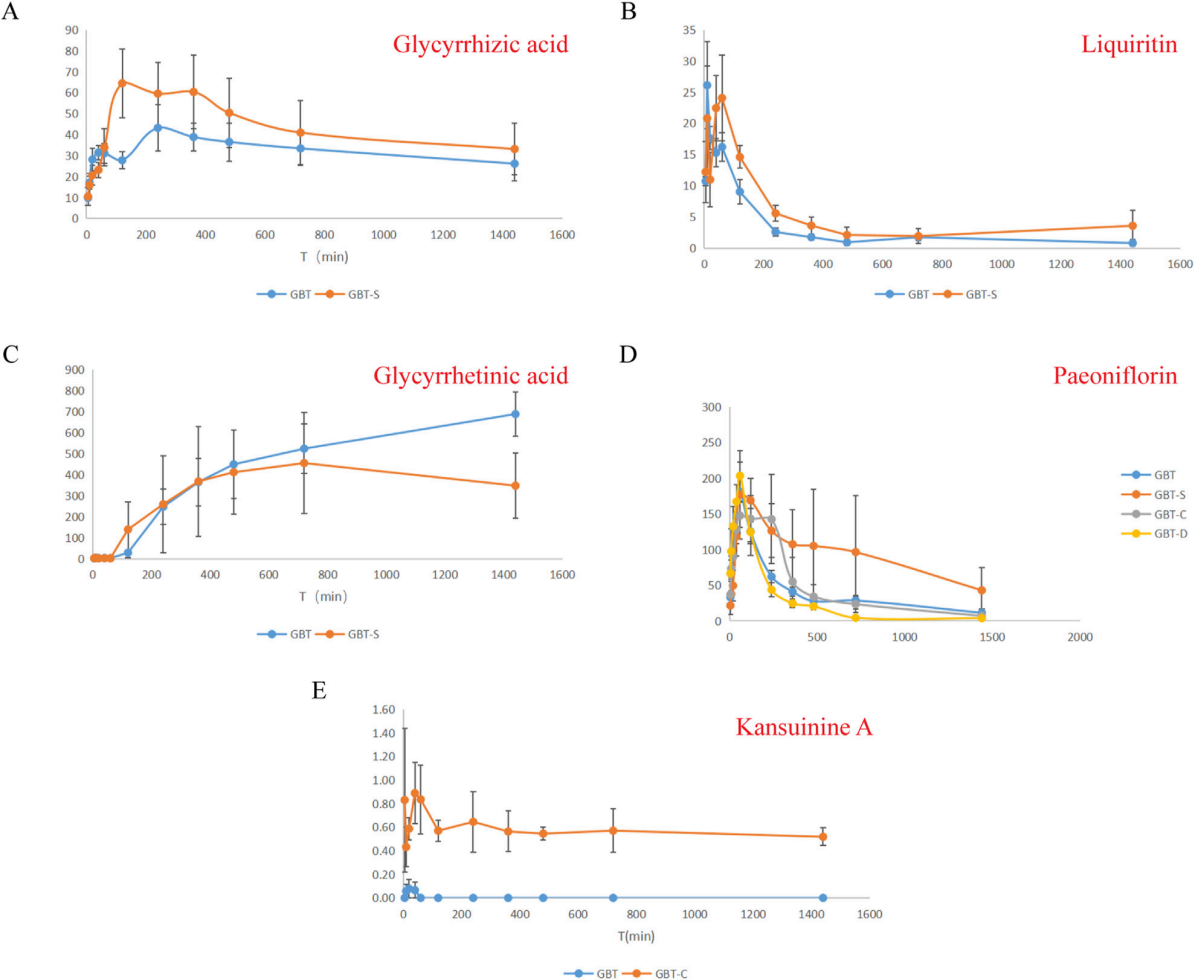
Plasma concentration-time curves for glycyrrhizic acid **(A)**, liquiritin **(B)**, glycyrrhetinic acid **(C)**, paeoniflorin **(D)**, kansuinine A **(E)**.

After the plasma samples were tested, the plasma concentration was calculated by substituting the standard curve of the day, and the concentration-time curves for the components were obtained by plotting the changes in plasma concentrations (C) over time (T) (Figure 1). The pharmacokinetic parameters of the non-compartmental analysis model were calculated using the R language, and the differences between the groups were compared using an analysis of variance (ANOVA) or t-test. The results are shown in Table 3.

**TABLE 3:** Pharmacokinetic parameters of five components in MA rats (n = 6), *P < 0.05 vs. GBT.

Compound	Group	Cmax/ng·mL-1	Tmax/h	T1/2/h	AUC_0-t_/ng•h•mL-1
Paeoniflorin	GBT	183.9 ± 34.59	0.93 ± 0.15	10.89 ± 8.82	930.99 ± 198.80
	GBT-S	203.4 ± 87.91	1.60 ± 0.55	12.08 ± 10.21	1294.37 ± 622.39
	GBT-C	189.33 ± 124.88	1.61 ± 1.25	12.71 ± 17.38	1106.22 ± 879.41
	GBT-D	209.17 ± 84.37	1.11 ± 0.46	4.21 ± 3.03	709.88 ± 311.29
Liquiritin	GBT	27.02 ± 14.39	0.37 ± 0.36	6.58 ± 4.47	66.89 ± 26.41
	GBT-S	30.15 ± 18.93	1.19 ± 0.70^*^	11.54 ± 13.19	117.12 ± 87.93
Glycyrrhizic acid	GBT	49.34 ± 17.66	10.00 ± 8.49	17.97 ± 14.96	780.03 ± 368.48
	GBT-S	88.75 ± 29.60^*^	6.72 ± 8.87	9.87 ± 2.12	1026.32 ± 613.11
Glycyrrhetinic acid	GBT	696.9 ± 235.39	21.60 ± 5.37		10,936.175 ± 5,404.52
	GBT-S	303.83 ± 188.81^*^	16.33 ± 8.62		5,273.19 ± 3,556.06
Kansuinine A	GBT	0.00 ± 0.00	0.00 ± 0.00		0.00 ± 0.00
	GBT-C	1.58 ± 1.14^*^	5.29 ± 5.61		14.55 ± 2.35

According to the pharmacokinetic results (Figure 1; Table 3), the addition of kansui significantly reduced the peak concentration (Cmax) of glycyrrhizic acid, while the peak time (Tmax) and half-life time (T1/2) showed an increasing trend, and the area under the curve (AUC) showed a decreasing trend. This indicates that kansui could reduce the metabolism and bioavailability of glycyrrhizic acid. However, the Cmax of glycyrrhetinic acid significantly increased, and the AUC also had increased tread after adding kansui. As a result, adding kansui can enhance glycyrrhetinic acid’s bioavailability. Liquiritin’s Cmax remained unchanged after adding kansui, but its Tmax was considerably reduced from 1.19 to 0.37 h. Nonetheless, a declining tendency was observed in both T1/2 and AUC. It is suggested that the addition of kansui could increase liquiritin’s metabolism while decreasing its bioavailability. No kansuinine A was found after adding liquorice, suggesting that liquorice may lessen the hazardous substance’s ability to leach from kansui. The addition of liquorice or/and kansui did not significantly alter the metabolism of paeoniflorin.

Based on the above results, we hypothesize that the drug metabolism of kansui and liquorice could mutually influence. In particular, liquorice could decrease the solubility of kansuinine A, while kansui may decrease the metabolism and bioavailability of glycyrrhizic acid while concurrently increasing the bioavailability of glycyrrhetinic acid.

### 3.2 Network pharmacological results of GBD

#### 3.2.1 Active compounds and potential targets of GBD

The chemical composition information of five herbs in GBD [GS (kansui), GC (liquorice), BS (radix paeoniae alba), BX (pinellia ternata), and FM (honey)] was searched in TCMSP, ETCM, the Database of Traditional Chinese Medicine and Chemistry, and published literature. The chemical compositions of each herb in the GBD are summarised in Supplementary Material 1 *(Worksheet 1: GBD’s chemical compositions)*. The active chemical compositions were screened by Swiss ADME according to Section 2.2.1. After merging, 278 active ingredients were obtained (Supplementary Material 1 *; Worksheet 2: Active chemical compositions)*. The target of action of each active chemical composition was predicted by Swiss Target Prediction, and 1,166 potential drug targets were obtained (Supplementary Material 1 *; Worksheet 3: Chemical composition targets)*. The number of active chemical compositions and potential drug targets is shown in Supplementary Table 13.

**FIGURE 2:**
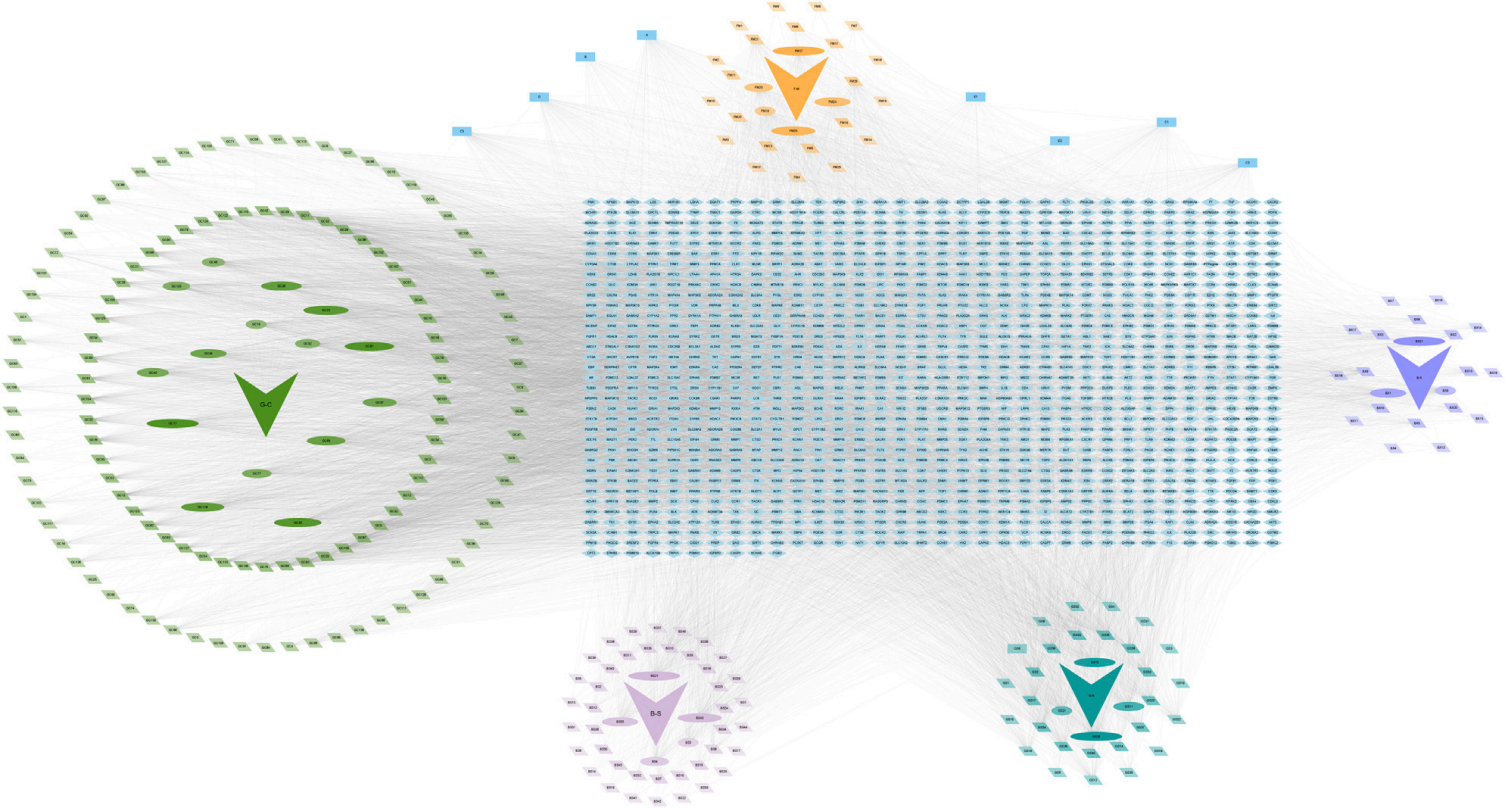
Herb-compound-target network of GBD. The arrows represent traditional herbs, with light green for GC, orange for FM, dark purple for BX, dark green for KS, and light purple for BS. The herb is at the center, with its active compounds on the periphery. The closer the compounds are to the center, the larger their shape, the darker their color, and the more lines connecting them to the herb. The dark blue rectangles represent the common components of two or more herbs. The light blue rectangular matrix in the middle represents the predicted drug therapeutic target genes of the active components.

To explain the relationship scientifically and reasonably between active components and targets, a herb-compound-target network was built and visualised using Cytoscape 3.10.1 (Figure 2), and the graphing data are shown in Supplementary Material 1 *(Worksheet 4: Graphing data for* Figure 2 *)*. The relevant information between nodes is presented in Supplementary Material 1 *(Worksheet 5: Node information)*. According to the number of undirected edges between the active components and the potential drug targets, the top 10% active components of each herb were selected (Supplementary Table 14).

#### 3.2.2 MA-related targets

We screened MA-related disease targets by searching disease-related databases and analysing MA-related GEO datasets. Firstly, the MA-related targets were searched in Drugbank, GeneCard, OMIM, PharmGKB, and TTD databases with “malignant ascites,” “malignant effusion,” and “malignant hydrops” as keywords. GeneCard used a three-fold median as the screening condition. The number of MA-related targets screened in each database is shown in Supplementary Table 15, and 753 MA-related targets were obtained after deletion of duplicate genes. The MA-related targets screened for each database are shown in Supplementary Material 1 *(Worksheet 6: MA-related targets)*.

The same keywords were used to screen MA-related datasets in the GEO datasets. According to the study requirements, three datasets (GSE73168, GSE39204, and GSE15831) were selected, and 1,135 DEGs were identified (Supplementary Material 1 *: Worksheet 6: MA-related targets)*. The number of DEGs screened for each dataset is shown in Supplementary Table 16. The analysis data results of the above three datasets are shown in Supplementary Material 1 *(Worksheet 7: DEGs in GEO database)*, and the results are visualised as volcano plots (S1) in (Supplementary Figure 3).

The MA-related targets screened from five databases and the DGEs screened from the GEO database were merged to obtain 1,866 MA-related targets, and 196 intersection targets were obtained after intersecting with 1166 potential drug targets (Supplementary Figure 4). The intersection targets were the key targets of GBD in the treatment of MA. The 196 intersection targets are shown in Supplementary Material 1 *(Worksheet 8: Intersection targets)*.

#### 3.2.3 Preliminary GO and KEGG analysis of intersecting targets

**FIGURE 3:**
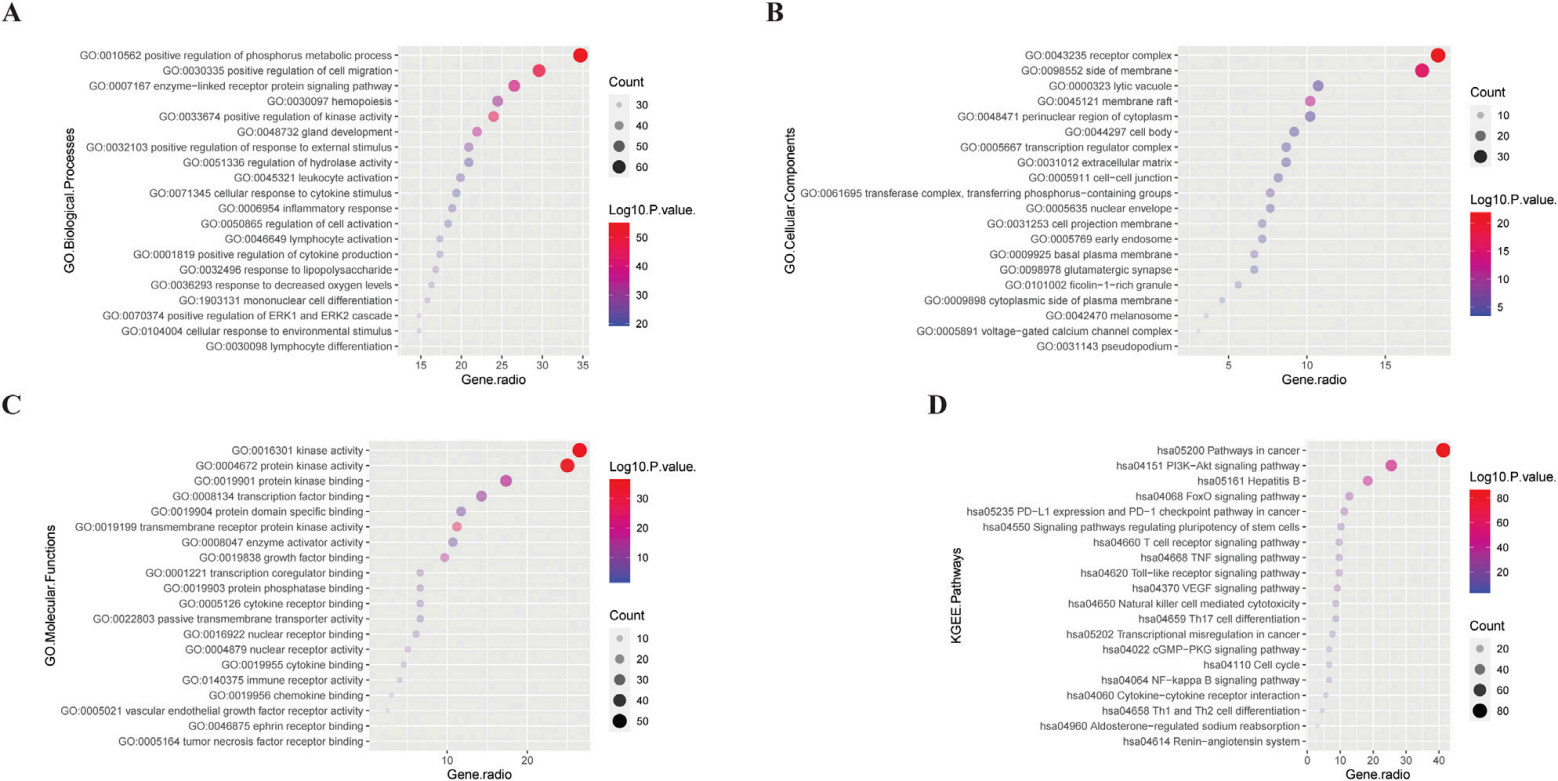
Bubble plots of GO (GO BP **(A)**, GO CC **(B)**, GO MF **(C)**), and KEGG **(D)** analysis results for 196 intersection genes. The larger the bubble, the more intersection genes are enriched in this item; the smaller the bubble, the fewer intersection genes. The redder the colour and the further away from the X-axis, the smaller the P-value of this entry; the opposite is even bigger.

The 196 intersection genes were imported into the Metascape database for enrichment analysis, and the filtering conditions were according to the default options. The detailed results of the GO and KEGG enrichment analyses are shown in Supplementary Material 1 *(Worksheet 9: GO and KEGG analyses)*. The results related to the research directions were visualised using the “ggplot2” package in R language, as shown in Figure 3.

In terms of BP, GBD may be related to the activation of various cell functions and the differentiation of immune cells. In terms of CC, GBD may be associated with a variety of membrane structures. In terms of cell biological functions, GBD may be related to the regulation of cell activation, differentiation, migration, and stress.

In the KEGG pathway, GBD was related to cancer pathways (vascular endothelial growth factor), tumor-related immune cells [such as T, natural killer (NK), and Th17 cells], inflammation-related pathways [such as toll-like receptors, nuclear factor kappa B (NF-κB), and tumor necrosis factor (TNF)], fluid metabolism [renin angiotensin aldosterone system (RAAS), natriuretic peptide (NP) system], and cell cycles.

#### 3.2.4 Key functional modules of GO BP

**FIGURE 4:**
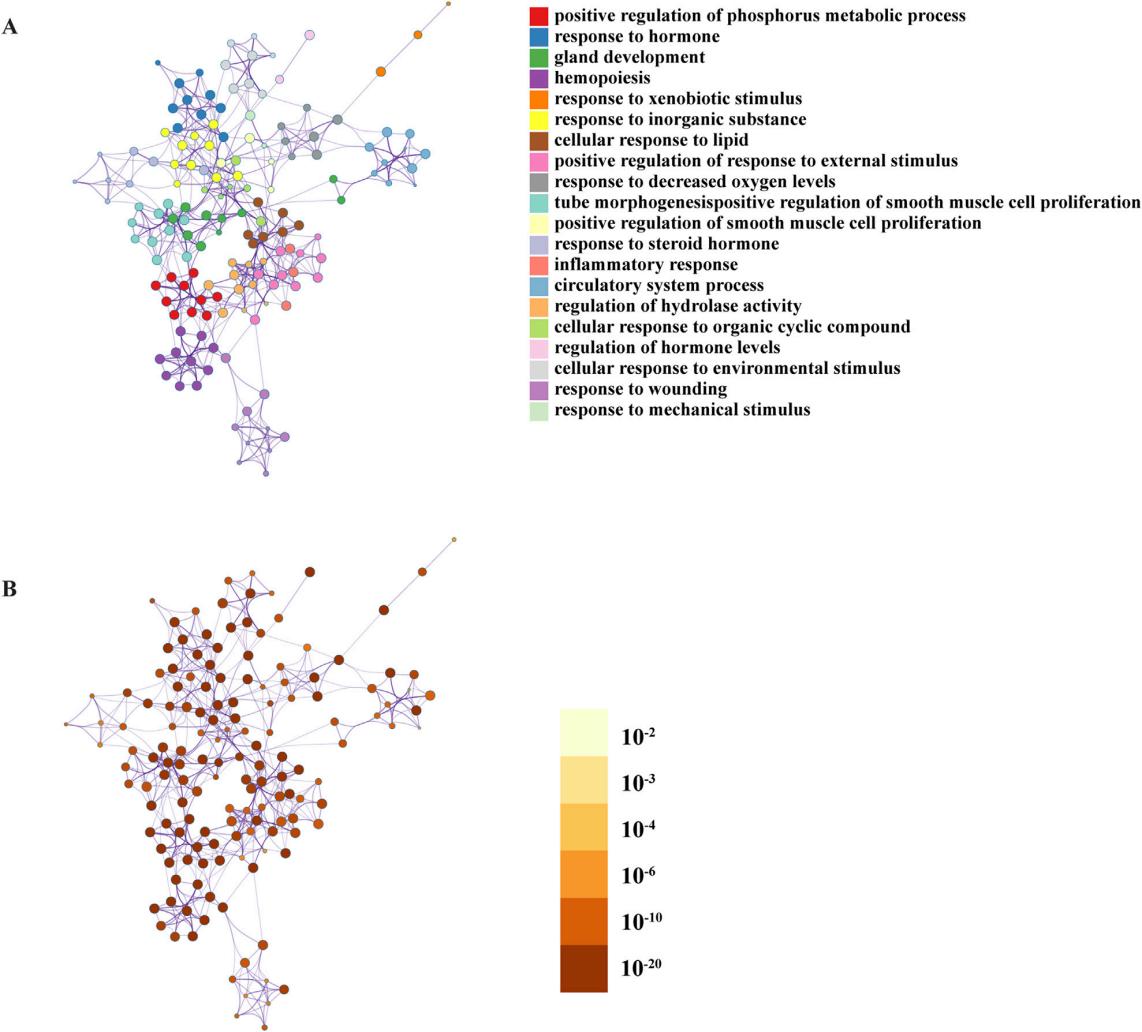
Network of enriched terms under the GO Biological Processes: **(A)** coloured by cluster ID, where nodes that share the same cluster ID are typically close to each other; **(B)** coloured by p-value, where terms containing more genes tend to have a more significant p-value.

In Metascape, to further capture the relationships between the terms of GO BP, a subset of enriched terms was selected and rendered as a network plot, where terms with a similarity >0.3 are connected by edges. We selected the terms with the best p-values from each of the 20 clusters, with the constraint that there were no more than 15 terms per cluster, and no more than 250 terms in total. The network was visualised using Cytoscape 5 (Figure 4; Table 4).

**TABLE 4:** Top 20 terms of Degree value under the GO Biological Processes.

GO	Category	Description	Count	%	Log10 (P)	Log10 (q)
GO:0010562	GO Biological Processes	Positive regulation of phosphorus metabolic process	68	34.69	−58.97	−55.09
GO:0009725	GO Biological Processes	Response to hormone	66	33.67	−54.49	−50.91
GO:0048732	GO Biological Processes	Gland development	43	21.94	−38.05	−35.19
GO:0030097	GO Biological Processes	Hemopoiesis	48	24.49	−35.09	−32.27
GO:0009410	GO Biological Processes	Response to xenobiotic stimulus	39	19.90	−32.85	−30.05
GO:0010035	GO Biological Processes	Response to inorganic substance	41	20.92	−31.56	−28.78
GO:0071396	GO Biological Processes	Cellular response to lipid	41	20.92	−31.26	−28.51
GO:0032103	GO Biological Processes	Positive regulation of response to external stimulus	41	20.92	−30.44	−27.73
GO:0036293	GO Biological Processes	Response to decreased oxygen levels	32	16.33	−28.58	−25.98
GO:0035239	GO Biological Processes	Tube morphogenesis	42	21.43	−28.16	−25.58
GO:0048661	GO Biological Processes	Positive regulation of smooth muscle cell proliferation	22	11.22	−27.23	−24.70
GO:0048545	GO Biological Processes	Response to steroid hormone	30	15.31	−27.21	−24.69
GO:0006954	GO Biological Processes	Inflammatory response	37	18.88	−25.86	−23.41
GO:0003013	GO Biological Processes	Circulatory system process	35	17.86	−25.21	−22.78
GO:0051336	GO Biological Processes	Regulation of hydrolase activity	41	20.92	−24.83	−22.40
GO:0071407	GO Biological Processes	Cellular response to organic cyclic compound	34	17.35	−24.56	−22.16
GO:0010817	GO Biological Processes	Regulation of hormone levels	35	17.86	−24.11	−21.74
GO:0104004	GO Biological Processes	Cellular response to environmental stimulus	29	14.80	−24.05	−21.69
GO:0009611	GO Biological Processes	Response to wounding	32	16.33	−24.01	−21.66
GO:0009612	GO Biological Processes	Response to mechanical stimulus	25	12.76	−23.48	−21.16

We found that GBD may be related to the following: (1) stress responses such as xenobiotic, decreased oxygen, environmental, mechanical, and injury stimulus; (2) cell exert functional processes, such as the regulation of hydrolytic enzyme activity and positive phosphorus metabolism; (3) inflammatory response; (4) hormone regulation; (5) gland development; (6) circulatory system; (7) regulation of smooth muscle cell proliferation; (8) hematopoiesis; and other BP.

**FIGURE 5:**
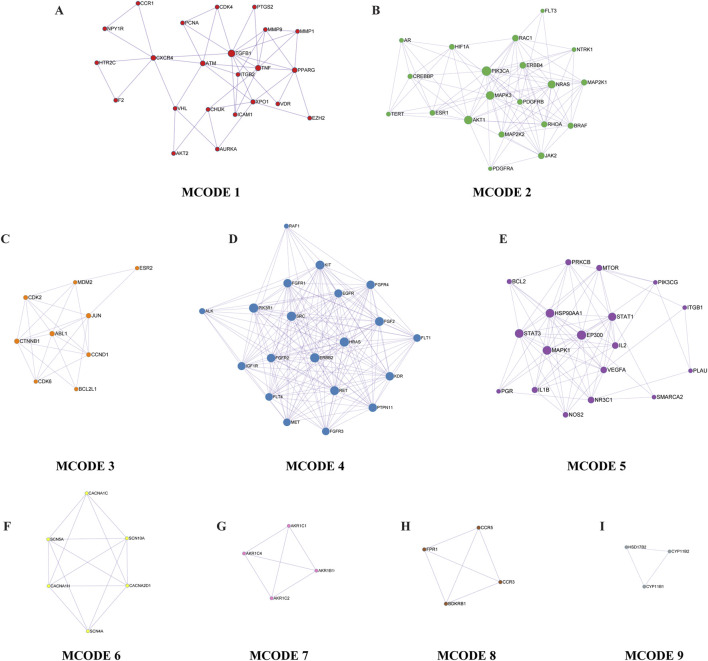
Key functional modules. From A to I are MCODE 1 through 9, in that order. Each small dot in the module represents the enriched gene, with the gene’s name marked next to the dot.

Further, the Metascape database used the Molecular Complex Detection (MCODE) algorithm to identify tightly connected network components, and classified the intersection genes into nine key functional modules (Figure 5). The analysis of the modules found that the intersection gene roles were mainly focused on transmembrane cell motility, protein phosphorylation, cell migration, cell cycle regulation, hormone synthesis and metabolism, inflammatory response and Ga^2+^ transmembrane motility, and other BP. The results of the enrichment of the GO BP of each module are shown in Table 5.

**TABLE 5:** The results of the enrichment of the GO Biological Processes of each module.

MCODE	GO	Description	Log10 (P)
My List	GO:0010562	Positive regulation of phosphorus metabolic process	−60.0
	GO:0045937	Positive regulation of phosphate metabolic process	−60.0
	GO:0042327	Positive regulation of phosphorylation	−57.3
MCODE 1	GO:0045834	Positive regulation of lipid metabolic process	−10.9
	GO:0030335	Positive regulation of cell migration	−9.7
	GO:2000147	Positive regulation of cell motility	−9.6
MCODE 2	GO:0007169	Transmembrane receptor protein tyrosine kinase signalling pathway	−37.4
	GO:0007167	Enzyme-linked receptor protein signalling pathway	−34.0
	GO:0042327	Positive regulation of phosphorylation	−30.4
MCODE 3	GO:0007169	Transmembrane receptor protein tyrosine kinase signalling pathway	−21.6
	GO:0042327	Positive regulation of phosphorylation	−20.9
	GO:0010562	Positive regulation of phosphorus metabolic process	−20.2
MCODE 4	GO:0009725	Response to hormone	−11.4
	GO:0030335	Positive regulation of cell migration	−10.7
	GO:2000147	Positive regulation of cell motility	−9.5
MCODE 5	GO:0045786	Negative regulation of cell cycle	−9.5
	GO:0007346	Regulation of mitotic cell cycle	−8.7
	GO:0045930	Negative regulation of mitotic cell cycle	−8.5
MCODE 6	GO:0098703	Calcium ion import across plasma membrane	−17.6
	GO:0097553	Calcium ion transmembrane import into cytosol	−14.7
	GO:0098659	Inorganic cation import across plasma membrane	−14.6
MCODE 7	GO:0007204	Positive regulation of cytosolic calcium ion concentration	−9.0
	GO:0006954	Inflammatory response	−7.0
	GO:0019932	Second-messenger-mediated signalling	−5.8
MCODE 8	GO:0044597	Daunorubicin metabolic process	−14.4
	GO:0044598	Doxorubicin metabolic process	−14.2
	GO:0030638	Polyketide metabolic process	−14.2
MCODE 9	GO:0120178	Steroid hormone biosynthetic process	−9.1
	GO:0042446	Hormone biosynthetic process	−8.6
	GO:0006694	Steroid biosynthetic process	−7.3

#### 3.2.5 Key pathways in KEGG

The enrichment results of KEGG pathways analysed by Metascape were imported into the KEGG database to understand their biological significance [the biological significance of each pathway is detailed in Supplementary Material 1 *(Worksheet 10: KEGG-Biological significance)]*.

According to the biological significance of the pathways, they were divided into 11 categories (Table 6), including different disease pathways, intracellular signalling pathways, intercellular signalling pathways, immunoinflammation-related pathways, cancer-related pathways, microbial infectious disease, endocrine-related pathways, nervous system related pathways, circulatory system related pathways, energy metabolism-related pathways, and others. Among them, 10 “Immunoinflammation-related” pathways were related to cellular immunity, of which four were related to T cells, indicating the importance of T cell immunity. In addition, NK cell related pathways are also included.

**TABLE 6:** Classification of KEGG pathways.

Classification	Number	Terms
Different disease pathways	23	hsa05215, hsa05218, hsa05224, hsa05212, hsa05211, hsa05223, hsa05226, hsa05210, hsa05214, hsa05220, hsa05225, hsa05219, hsa05221, hsa05213, hsa05216, hsa04934, hsa05222, hsa04932, hsa05321, hsa05323, hsa04936, hsa05034, hsa05010
Intracellular signalling pathways	33	hsa04151, hsa04010, hsa04015, hsa04014, hsa04066, hsa04510, hsa04068, hsa04218, hsa04210, hsa04810, hsa04012, hsa04370, hsa04072, hsa04071, hsa04150, hsa04024, hsa04140, hsa04022, hsa04380, hsa04020, hsa04630, hsa04213, hsa04115, hsa04110, hsa04350, hsa04217, hsa04141, hsa04144, hsa04070, hsa04215, hsa04310, hsa04137, hsa04390
Intercellular signalling pathways	6	hsa04371, hsa04540, hsa04620, hsa04668, hsa04520, hsa04530
Immunoinflammation-related pathways	25	hsa04062, hsa04660, hsa04664, hsa04662, hsa04650, hsa04613, hsa04666, hsa04625, hsa04621, hsa04657, hsa04670, hsa04659, hsa04658, hsa01523, hsa04623, hsa04064, hsa04750, hsa04145, hsa04514, hsa04060, hsa05332, hsa04672, hsa04612, hsa05330, hsa04610
Cancer-related pathways	13	hsa05200, hsa05205, hsa01521, hsa05230, hsa05206, hsa05207, hsa05235, hsa05208, hsa05231, hsa05203, hsa04550, hsa05202, hsa04916
Microbial infectious disease	32	hsa05167, hsa05161, hsa05163, hsa05166, hsa05165, hsa05170, hsa05132, hsa05145, hsa05135, hsa05142, hsa05169, hsa05131, hsa05171, hsa05140, hsa05130, hsa05168, hsa05100, hsa05143, hsa05150, hsa05120, hsa05020, hsa03250, hsa05416, hsa04061, hsa05164, hsa05160, hsa05162, hsa05152, hsa05133, hsa05144, hsa05146, hsa05134
Endocrine-related pathways	32	hsa01522, hsa04926, hsa04919, hsa04917, hsa04915, hsa04935, hsa04921, hsa04929, hsa04912, hsa04914, hsa04933, hsa04928, hsa04910, hsa04114, hsa04931, hsa04930, hsa04960, hsa00140, hsa04940, hsa04922, hsa04713, hsa04972, hsa04970, hsa04925, hsa04927, hsa04911, hsa04913, hsa04924, hsa04614, hsa04976, hsa00790, hsa04961
Nervous system related pathways	19	hsa04722, hsa04725, hsa05022, hsa04726, hsa04730, hsa04720, hsa04360, hsa05017, hsa04080, hsa05014, hsa05012, hsa05016, hsa04728, hsa04723, hsa05031, hsa04724, hsa04727, hsa05032, hsa05030
Circulatory system related pathways	8	hsa04270, hsa04261, hsa05417, hsa05418, hsa05415, hsa05410, hsa05414, hsa05412
Energy metabolism-related pathways	6	hsa04152, hsa04211, hsa04920, hsa04973, hsa04923, hsa04714
Else	3	hsa01524, hsa04611, hsa04640

One KEGG pathway to which other pathways are related can be found in the KEGG database. Accordingly, we searched the relationships among enriched pathways obtained in the Metascape website. We imported the interrelations between pathways into Cytescape 3.9.1 and calculated the interrelations between nodes. The imported data *(Worksheet 11: KEGG-Interrelationships)* and the results *(Worksheet 12: KEGG-relationship results)* are shown in Supplementary Material 1.

**FIGURE 6:**
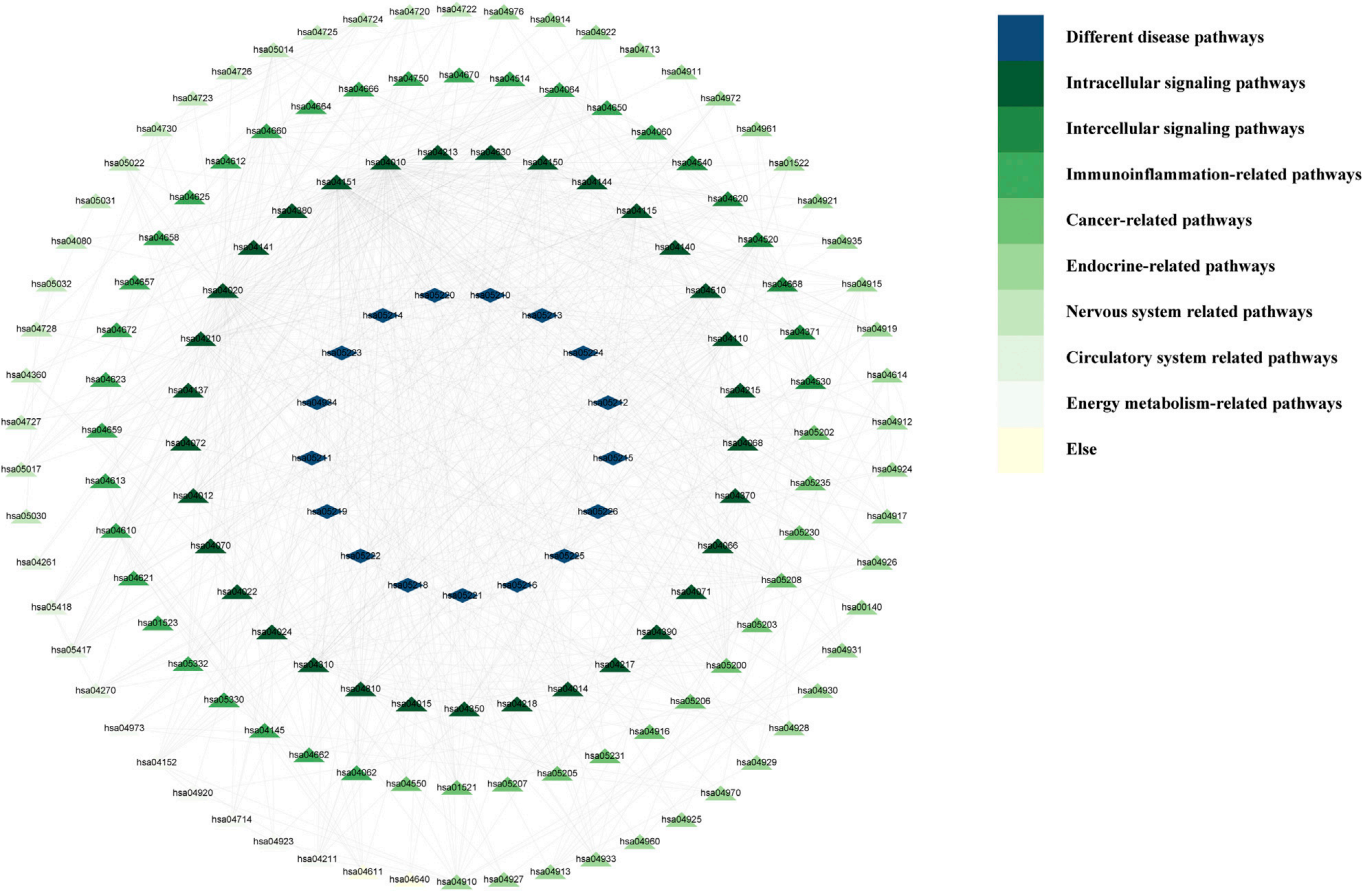
The correlation network between KEGG pathways.

Then, the interrelationships between KEGG pathways were visualised in Cytoscape 3.10.1 (Figure 6). In this figure, there are no pathways associated with the following pathways: hsa04080, hsa00140, hsa04640, hsa04061, hsa04060, and hsa00790. In addition, we deleted the pathways in the “Microbial infectious disease” and those unrelated to ascites in the “Different disease pathways.” The inner circle layer, including “Intracellular signalling pathways,” “Intercellular signalling pathways,” “Immunoinflammation-related pathways,” and “Cancer-related pathways” had the most connectivity.

The top 20 KEGG pathways were screened according to the number of connections; that is, the degree values (Table 7). According to the analysis of the results, we learned that the most important pathways are mainly concentrated in the pathways related to apoptosis, immunity, inflammation, cell cycle, transcription, cell migration, and autophagy.

**TABLE 7:** The top 20 KEGG pathways.

KEGG number	Description	Degree	Betweenness centrality	Clustering coefficient	Clustering coefficient	Neighbourhood connectivity
hsa04010	MAPK signalling pathway	108	0.263	0.658	0.077	12.423
hsa04210	Apoptosis	83	0.162	0.595	0.089	13.467
hsa04151	PI3K-Akt signalling pathway	82	0.096	0.582	0.108	14.288
hsa04020	Calcium signalling pathway	72	0.182	0.588	0.056	10.912
hsa04110	Cell cycle	46	0.032	0.516	0.166	16.289
hsa04620	Toll-like receptor signalling pathway	41	0.035	0.509	0.151	16.462
hsa04115	p53 signalling pathway	38	0.019	0.494	0.173	16.861
hsa04630	JAK-STAT signalling pathway	37	0.023	0.510	0.175	17.500
hsa04064	NF-kappa B signalling pathway	36	0.022	0.501	0.168	17.903
hsa04150	mTOR signalling pathway	32	0.013	0.474	0.170	16.034
hsa04310	Wnt signalling pathway	31	0.020	0.469	0.171	15.556
hsa04810	Regulation of actin cytoskeleton	28	0.018	0.474	0.129	14.538
hsa04660	T cell receptor signalling pathway	26	0.026	0.494	0.149	18.750
hsa04350	TGF-beta signalling pathway	25	0.023	0.489	0.170	17.840
hsa04144	Endocytosis	25	0.029	0.411	0.078	9.682
hsa04610	Complement and coagulation cascades	25	0.013	0.407	0.111	9.565
hsa04510	Focal adhesion	22	0.007	0.459	0.242	22.833
hsa04024	cAMP signalling pathway	21	0.021	0.479	0.119	16.000
hsa04140	Autophagy - animal	21	0.007	0.451	0.175	15.895
hsa04012	ErbB signalling pathway	20	0.008	0.452	0.228	18.765
hsa04520	Adherens junction	20	0.004	0.460	0.343	26.867

#### 3.2.6 PPI network of intersecting targets

**FIGURE 7:**
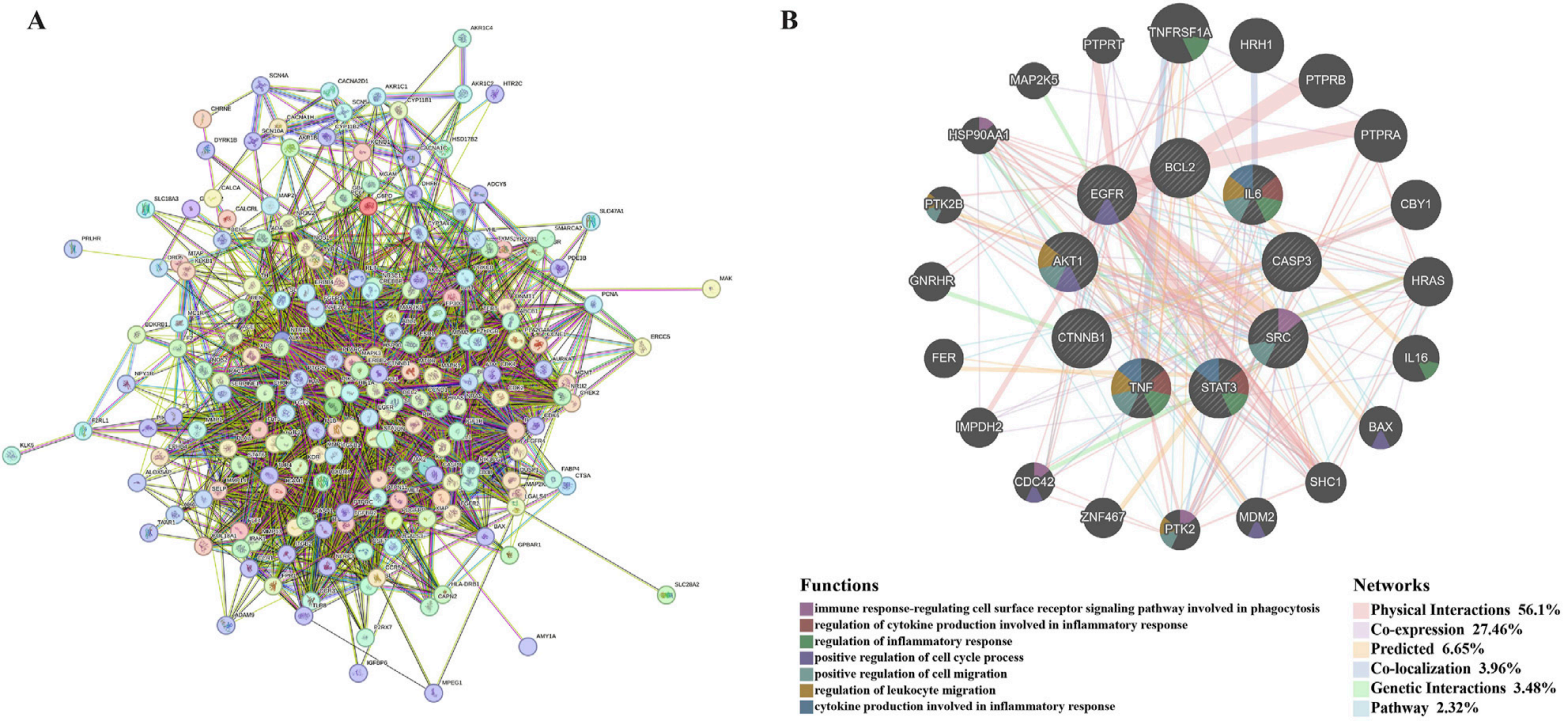
Hub genes prediction. **(A)** is the PPI network obtained after importing the intersection genes into STRING online database. **(B)** is the co-expression network of hub genes. Hub genes and their co-expression genes were analysed via GeneMANIA. The hub genes were located in the inner circle, while the predicted genes were in the outer circle. Each gene is represented by a circle, and the different colors in the circle indicate the function of the gene. The gene size represents the strength of interactions. The inter-gene connection lines represent the types of gene-gene interactions, and the line color represents the types of interactions.

The intersection genes obtained in 3.2.2 were imported into STRING (https://string-db.org/), and “Organisms” was selected for “*H. sapiens*” to make a PPI network (Figure 7A).

#### 3.2.7 Selection and analysis of hub genes

The PPI network of intersection genes obtained in STRING was imported into Cytoscape 3.10.1, and the hub genes were screened using the cytoHubba plug-in. We selected six algorithms (Degree, MCC, MNC, EPC, Radiality, and Closeness) to evaluate and screen hub genes, and each algorithm took the top 10. The screening details of the hub genes are shown in Supplementary Material 1 (*Worksheet 13: Gene screen*). The Draw Venn Diagram (https://bioinformatics.psb.ugent.be/webtools/Venn/) was used to obtain nine hub genes (Supplementary Figure 5), which were AKT1, STAT3, BCL2, IL6, TNF, CTNNB1, EGFR, SRC, and CASP3.

GeneMANIA was used to predict functionally similar genes of hub genes. We obtained 20 similar genes of hub genes (Figure 7B). The hub genes were located in the inner circle, while the predicted genes were in the outer circle. In this network, the Physical Interactions Co-expression, Predicted, Co-localisation, Genetic Interactions, Pathway values were 56.1%, 27.46%, 6.65%, 3.96%, 3.48%, and 2.32%, respectively. These genes are related to the regulation of immune-inflammatory response, cell cycle, and cell migration.

In summary, the key genes were obtained after the intersection of the active ingredient targets of GBD and the MA-related targets. Then, KEGG and GO enrichment analysis of key genes showed that the key genes were closely related to inflammation and immunity. Through the plugin-in cytoHubba in Cytoscape 3.9.1, six algorithms were used to screen the hub genes in the intersection genes, and the hub genes were imported into GeneMANIA to obtain functionally similar genes, which also verified that the functions of hub genes were closely related to inflammation and immunity. Among them, T and NK cell immunity was mentioned several times in the above prediction.

### 3.3 Experimental verification of the efficacy of GBD with or without kansui-liquorice in MA rats

#### 3.3.1 Effects of GBD with or without kansui-liquorice on cellular immunity of MA rats

In our previous study, we have demonstrated that GBD has efficacy in improving cellular immunity in MA rats (Huo et al., 2024). Combined with the results of network pharmacology prediction in this study, we believe that the efficacy of GBD in regulating immune function is extremely important in the treatment of MA. What role do kansui and Liquorice play in the immune regulation of GBD? Therefore, we conducted experiments to verify the differences in the effects of GBD with and without kansui and liquorice on its immune function.

Because the immunomodulatory effects of GBD have been proven (Huo et al., 2024), thus, the animal experiments in Sections 3.3.2, 3.3.3 were divided into only four groups of GBT, GBT-S, GBT-C, and GBT-D, and no CG, MG, or PMG. The gate step setting of flow cytometry is described in (Supplementary Figures 6–8).

##### 3.3.1.1 Effects of GBD with or without kansui-liquorice on cellular immunity in PB of MA rats

**FIGURE 8:**
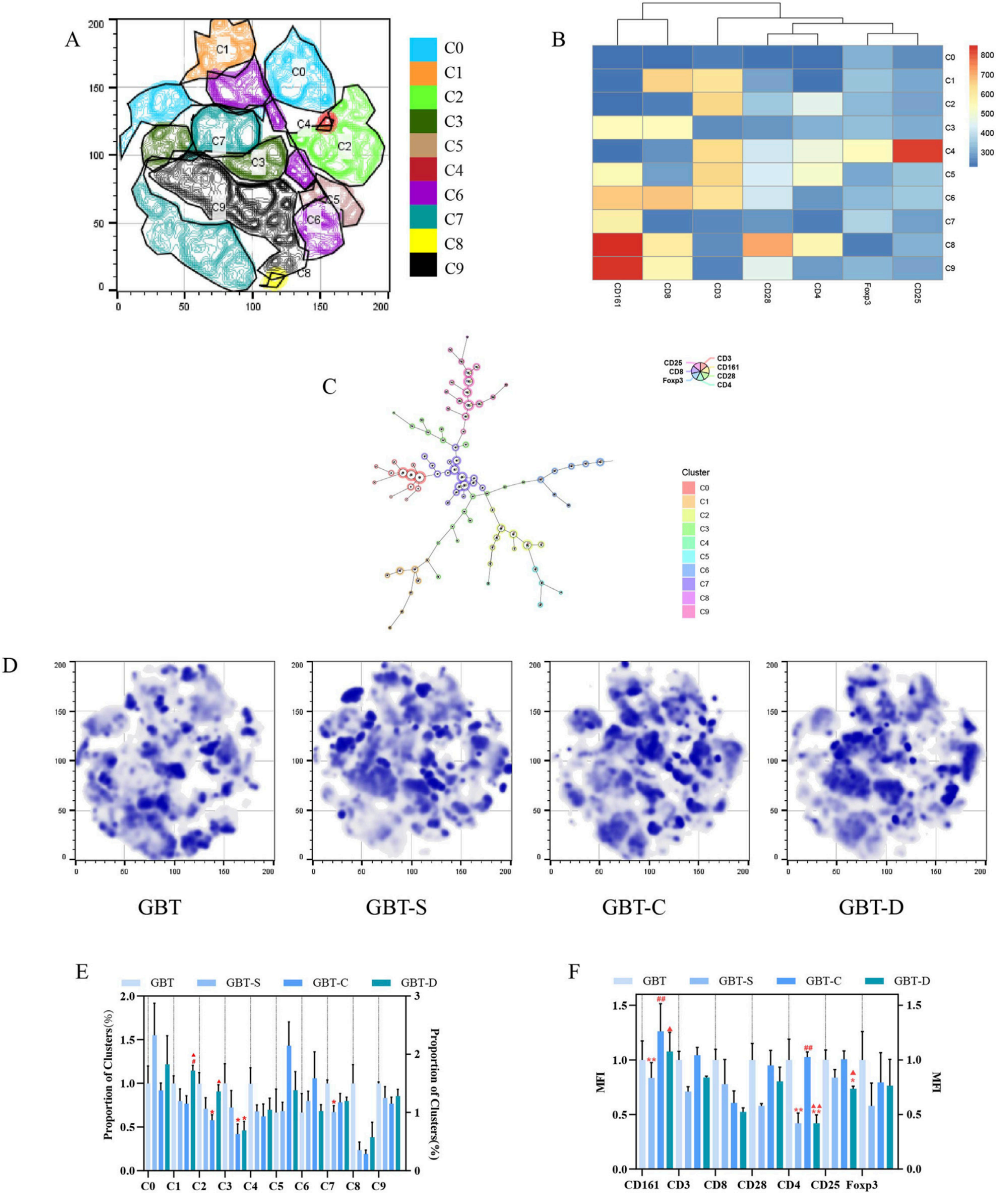
Immune cells in the PB. **(A)** The peripheral blood immune cells of each group were concatenated after descending to a unified standard, and then divided into 10 clusters by t-sne and flowsom dimensionality reduction analysis. Heat map **(B)** and dendrogram **(C)** of fluorescent expression of labelled proteins of each cluster. **(D)** The t-sne dimension reduction graph of experimental groups, from left to right, are GBT, GBT-S, GBT-C, and GBT-D. **(E)** The statistical graph of 10 clusters. **(F)**. The statistical graph of MFI of labelled proteins. The data are expressed as the mean ± SEM (n = 6). *P < 0.05, **P < 0.01 vs. GBT. ^#^ *P* < 0.01, ^##^ *P* < 0.01 vs. GBT-S. ^▲^ *P* < 0.05, ^▲▲^ *P* < 0.01 vs. GBT-C.

We analysed the effects of GBD with or without kansui-liquorice on immune cells (T and NK cells) in PB of MA rats, to explore the mechanism of GBD on cellular immunity. The PB immune cells of each group were concatenated after descending to a unified standard, and then divided into 10 clusters (Figure 8A) by t-sne and flowsom dimensionality reduction analysis. Each experimental group’s t-sne graph is shown in Figure 8D. The expression and proportion of labelled fluorescent proteins of the 10 clusters are shown in a heat map (Figure 8B), dendrogram (Figure 8C), and Table 8.

**TABLE 8:** The expression and proportion of labelled fluorescent proteins in the 10 clusters of PB.

Cluster	Fluorescent protein expression	Proportion (%)
C0	All (−)	14.3
C1	CD3 (+)CD8 (+)	6.17
C2	CD3 (+)CD4 (+)	11.9
C3	CD8 (+)CD161 (+)	10.2
C4	CD3 (+)CD4 (+)CD25 (+)Foxp3 (+)	0.36
C5	CD3 (+)CD4 (+)CD161 (+)	2.79
C6	CD3 (+)CD8 (+)CD161 (+)	14.2
C7	CD161 (+)	22.6
C8	CD4 (+)CD8 (+)CD28 (+)CD161 (+)	0.18
C9	CD8 (+)CD28 (+)CD161 (+)	19.2

According to the results, the types of immune cell subsets in PB were complex. In addition to C0 (none of the labelled proteins were expressed), there were five clusters accounting for more than 10%, followed by C7 (22.6%), C9 (19.2%), C6 (14.2%), C2 (11.9%), and C3 (10.2%). Except for C2, all the dominant clusters expressed CD161, indicating that NK cells were the most important immune cell subset in PB. Among them, the most co-expressed with CD161 was CD8. Notably, the CD4 T cell subset also plays an important role in PB (C2), but only a tiny fraction of regulatory T cells (Tregs, CD4 (+)CD25 (+)Foxp3 (+) cells) (C4).

A statistical analysis of the 10 clusters showed that, although there was no significant difference between the clusters of each experimental group, all the dominant clusters except C6 were at a high level in GBT (Figure 8E). The MFI (Figure 8F) analysis showed that GBT did not significantly increase the expression of CD161, but the expression of T cell-related proteins, such as CD3, CD8, CD4, and CD28 remained at a high level (Figure 8F).

**FIGURE 9:**
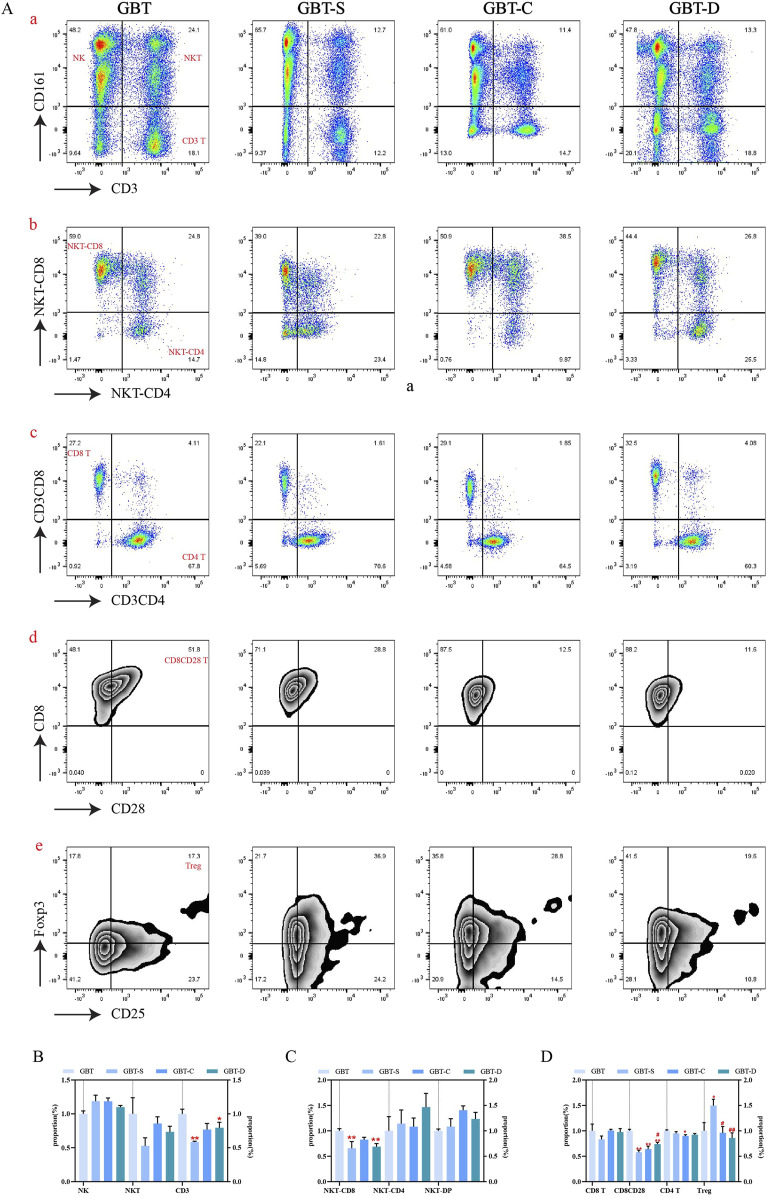
The peripheral blood immune cells of MA rats were manually analysed. **(A)** Includes scatter plots **(a–c)** and contour plots **(d, e)**. From left to right, GBT, GBT-S, GBT-C, and GBT-D were followed. **(a)** Distinguished NK cells and T cells, the upper left is NK cells, the upper right is NKT cells, and the lower right is T cells. **(b)** Expression of CD8 and CD4 in NKT cells. **(c)** Expression of CD8 and CD4 in T cells, the upper left is CD8 (+)CD4 (−) T cells, and the lower right is CD4 (+)CD8 (−) T cells. **(d)** Expression of CD28 in CD8 (+)CD4 (−) T cells. **(e)** Expression of CD25 and Foxp3 in CD4 (+) CD8 (−) T cells. **(B)** Statistical graph of **(A, a)**. **(C)** Statistical graph of **(A, b)**. **(D)** Statistical graph of **(A, c, d, e)**. The data are expressed as the mean ± SEM (n = 6). *P < 0.05, **P < 0.01 vs. GBT. ^#^ *P* < 0.01, ^##^ *P* < 0.01 vs. GBT-S.

We further manually analysed immune cells of PB, and found that there was no significant difference in NK cells and NKT cells among the experimental groups, but the proportion of T cells increased in the GBT (Figures 9A(a), B). In addition to this, the proportion of NKT expressing CD8 cells also relatively increased (Figures 9A(b), C). Although there was no significant difference between the groups in CD8 (+)CD4 (−) T cells and CD4 (+)CD8 (−) T cells (Figures 9A(c), D), the proportion of CD4 co-expressing CD25 and Foxp3 T cells at a low level (Figure 9A(e), D), and the proportion of CD8 co-expressing CD28 Tcells increased (Figures 9A(d), D) in the GBT. Those results correspond to the t-sne results.

In the other groups, the proportion of CD4 co-expressing CD25 and Foxp3 was also reduced (Figure 9A(e), D) in the GBT-C and GBT-D, but they had no obvious advantages in increasing the proportion of T cells (Figures 9A(a), B), NKT expressing CD8 T cells (Figures 9A(b), C) and CD8 co-expressing CD28 T cells (Figures 9A(d), D).

In conclusion, the immune environment in the PB of MA rats is complex, with a variety of immune cell subsets, the most important of which is NK cells. Compared with GBD without kansui-liquorice, GBD containing kansui-liquorice has no obvious advantage in increasing the proportion of NK cells in PB, but it can increase the co-expression of CD8 by NK cells. In addition, the advantage of GBD containing kansui-liquorice may be related to increasing the proportion of T cells, increasing the co-expression of CD28, and reducing the co-expression of CD25 and Foxp3. Thus, kansui-liquorice has a role in GBD in exerting peripheral blood immune function.

##### 3.3.1.2 Effects of GBD with or without kansui-liquorice on cellular immunity in TME of MA rats

**FIGURE 10:**
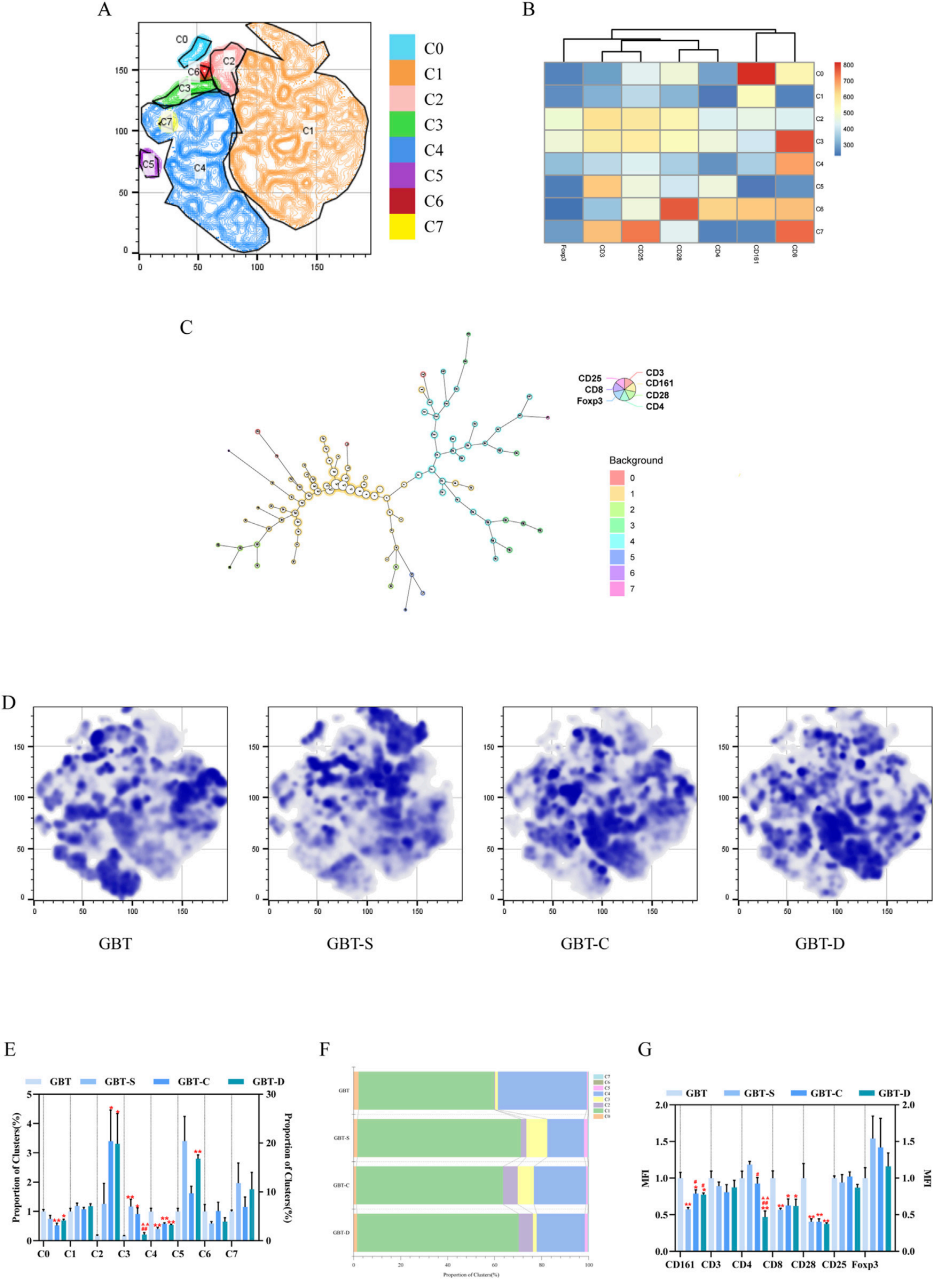
Immune cells in the TME. **(A)**. The immune cells in the TME of each group were concatenated after descending to a unified standard, and then divided into eight clusters by t-sne and flowsom dimensionality reduction analysis. Heat map **(B)** and dendrogram **(C)** of fluorescent expression of labelled proteins of each cluster. **(D)** The t-sne dimension reduction graph of experimental groups, from left to right, are GBT, GBT-S, GBT-C, and GBT-D. **(E)** Statistical graph of eight clusters. Percentage plot **(F)** of the proportion of eight clusters for each experimental group. **(G)** Statistical graph of MFI of labelled proteins. The data are expressed as the mean ± SEM (n = 6). *P < 0.05, **P < 0.01 vs. GBT. ^#^ *P* < 0.01, ^##^ *P* < 0.01 vs. GBT-S. ^▲▲^ *P* < 0.01 vs. GBT-C.

We further analysed the immune environment in the TME of MA rats, and the analysis method was similar to PB (Section 3.3.2.1). The t-sne graph of all experimental groups is shown in Figure 10A, and each experimental group’s graph is shown in Figure 10D. The immune cells in the TME were divided into eight clusters, and the expression and proportion of labelled fluorescent proteins in each cluster are shown in the heat map (Figure 10B), dendrogram (Figure 10C), percentage plot (Figure 10F), and Table 9.

**TABLE 9:** The expression and proportion of labelled fluorescent proteins in the eight clusters of TME.

Cluster	Fluorescent protein expression	Proportion (%)
C0	CD161 (+)CD8 (+)CD28 (+)	1.79
C1	CD161 (+)	63.0
C2	CD3 (+)CD25 (+)CD28 (+)Foxp3 (+)	4.20
C3	CD3 (+)CD4 (+)CD25 (+)CD28 (+)Foxp3 (+)	4.40
C4	CD8 (+)	25.2
C5	CD3 (+)CD4 (+)CD25 (+)	1.06
C6	CD161 (+)CD4 (+)CD8 (+)CD28 (+)	0.097
C7	CD3 (+)CD8 (+)CD25 (+)	0.27

Different from PB, the immune cell subsets in the TME were relatively simple, and C1 and C4 are the largest clusters, accounting for 78.2%, indicating that NK cells and CD8 cells were the two most crucial subsets in the TME. The immune cells expressing Foxp3 play an immunosuppressive role, and only C2 and C3 clusters were expressed. A statistical analysis of eight clusters showed that C0 and C4 clusters significantly increased (Figure 10E), and C2 and C3 clusters significantly decreased in the GBT (Figure 10E).

In the other groups, we found that the GBT-D could reduce the proportion of C3 cluster (Figure 10E), but there was no significant difference in other clusters among the three groups (Figure 10E).

**FIGURE 11:**
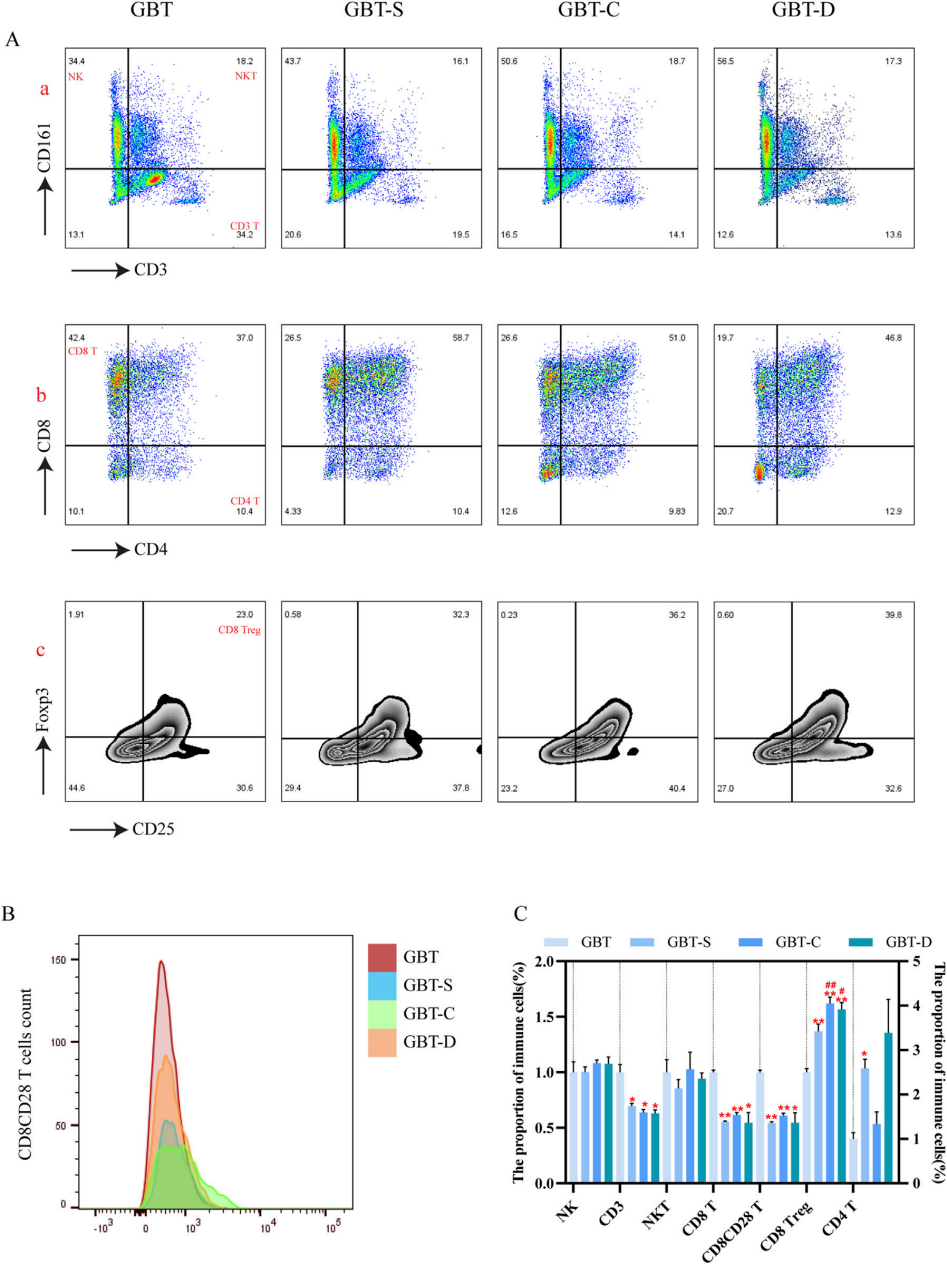
Immune cells in the TME were manually analysed in MA rats. From left to right, GBT, GBT-S, GBT-C, GBT-D were followed. **(A, a)** Distinguished NK cells and T cells, the upper left is NK cells, the upper right is NKT cells, and the lower right is T cells. **(A, b)** Expression of CD8 and CD4 in T cells. **(A, c)** Co-expression of CD25 and Foxp3 in CD8 T cells. The peak plot **(B)** of CD28 expression in CD8 (+)CD4 (−) T cells. **(C)** Statistical graphs of **(A, B)**. The data are expressed as the mean ± SEM (n = 6). *P < 0.05, **P < 0.01 vs. GBT. ^#^ *P* < 0.01, ^##^ *P* < 0.01 vs. GBT-S.

Consistent with the results of t-sne, we found that the proportion of the T cells (Figures 11A(a), C), CD8(+)CD4(−) T cells (Figure 11A(b)) and CD8(+) CD28(+) T cells (Figures 11B, C) in the GBT were significantly higher. In addition, the proportion of CD8 Treg cells (Figures 11A(c), C), which co-express CD25 and Foxp3 of CD8(+)CD4(-) T cells, was also reduced.

In the other groups, although the GBT-S was better than the other groups in reducing the proportion of CD8Treg cells (Figures 11A(c), C), there was no significant difference in the proportion of T cells (Figures 11A(a), C) and CD8(+)CD28(+)T (Figures 11B, C) cells between the three groups.

The above results further verified that GBD exerted a regulatory effect on T cell subsets in the TME of MA rats.

#### 3.3.2 Effects of GBD with or without kansui-liquorice on T cell development in MA rats

After analyzing immune cells in PB and TME, we found that GBD containing kansui-liquorice plays an important role in increasing T cell immunity. To investigate the effect of GBD with or without kansui-liquorice on the T cell development of MA rats, we further studied the proliferation and differentiation of T cells in the thymus and marrow.

7-AAD is a DNA dye that can be used to distinguish the cell cycle, but cannot distinguish the G0 and G1 phases. Ki-67 is a nuclear protein that is not detected in G0 phases, but is expressed from the G1 phase to the mitotic phase, and thus is often used as a proliferation marker. In this experiment, anti-CD3, anti-CD4, and anti-CD8 antibodies were used to identify T cell subsets in marrow and thymus, and 7-AAD and anti-Ki-67 double staining were used to distinguish the cell cycle of T cells.

##### 3.3.2.1 Effects of GBD with or without kansui-liquorice on T cells in the marrow of MA rats

**FIGURE 12:**
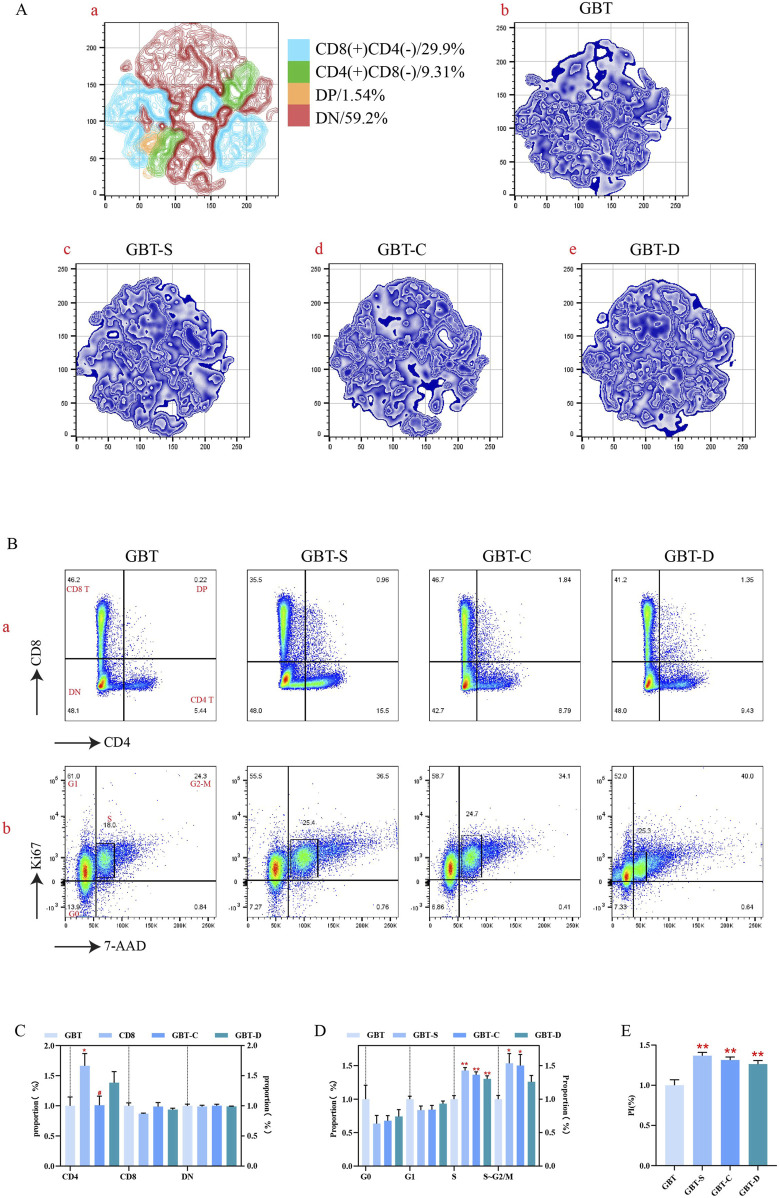
T cell differentiation and proliferation in the marrow. **(A, a)**. The T cells in the marrow of each group were concatenated after descending to a unified standard, and then divided into four subgroups by t-sne and flowsom dimensionality reduction analysis. The blue represents the CD8 (+)CD4 (−) T subgroup; green is the CD4 (+)CD8 (−) T subgroups; orange is the DP T subgroup; and red is the DN T subgroups. **(A, b–e)**. The t-sne dimension reduction graph of each experimental group, GBT, GBT-S, GBT-C, GBT-D in turn. **(B)** Manual analysis of T cell differentiation **(a)** and proliferation **(b)** in the marrow. From left to right are GBT, GBT-S, GBT-C, GBT-D. **(C, D)** are the statistical graphs of **(B, a, b)**. **(E)** The PI of marrow T cells of experimental groups. The data are expressed as the mean ± SEM (n = 6). *P < 0.05, **P < 0.01 vs. GBT, ^#^ *P* < 0.05 vs. GBT-S.

In this experiment, the preliminary analysis of T cell subsets in marrow was performed by t-sne dimensionality reduction analysis in all experimental groups after to the same standard (Figure 12A(a–e)). As can be seen, the largest T cell subgroups of marrow was double negative (DN), followed by CD8(+)CD4(-) T cells, CD4(+)CD8(-) T cells, and double positive (DP).

T cell subsets in the marrow of each group were analysed, and we found that the dominant subgroup in each experimental group was DN (Figure 12B(a)), and there was no significant difference during them (Figure 12C). Further analysis of the proliferation of T cell subgroups revealed that the marrow T cells in the S phase in GBT were reduced relative to the other groups (Figures 12B(b), D), and the proliferation index (PI) was also reduced (Figure 12E). However, there was no significant difference in the cell cycle (Figures 12B(b), D) and PI (Figure 12E) among the other groups.

In conclusion, there was no significant effect on T cell differentiation in the marrow of MA rats between GBD with or without kansui-liquorice, but GBD containing kansui-liquorice could reduce its proliferation.

##### 3.3.2.2 Effects of GBD with or without kansui-liquorice on T cells in the thymus of MA rats

**FIGURE 13:**
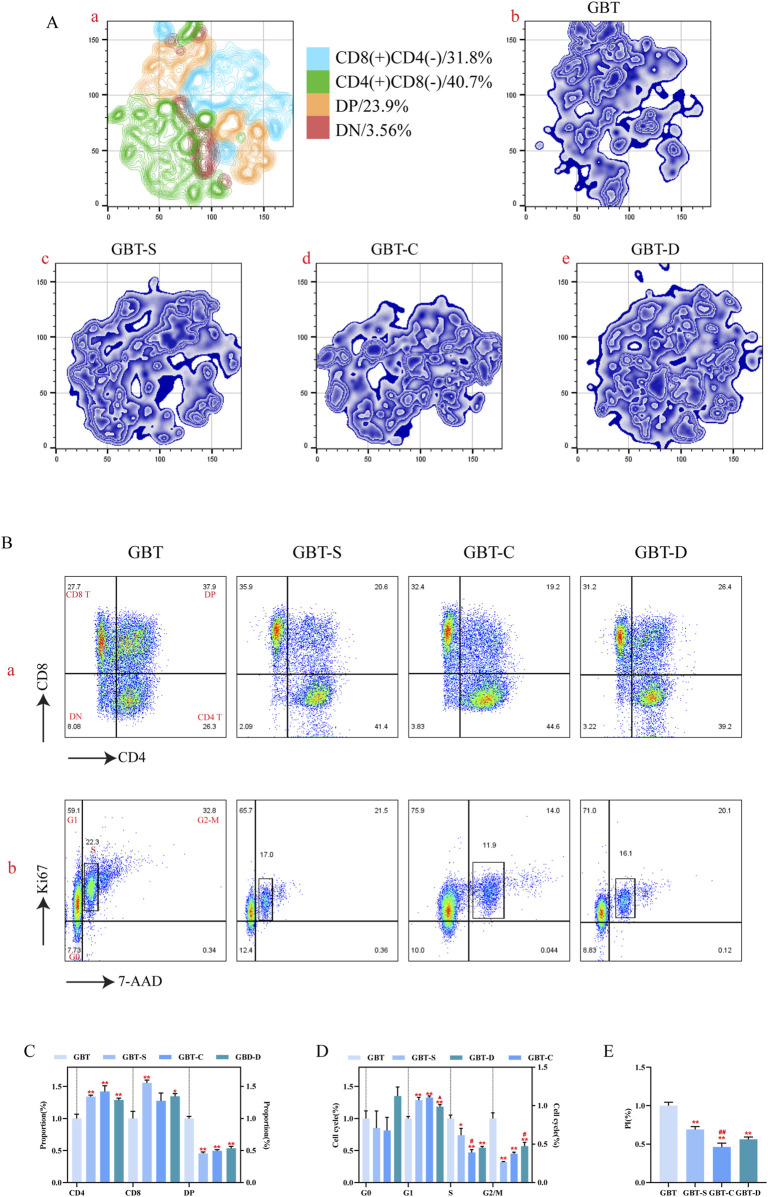
T cell differentiation and proliferation in the thymus. **(A, a)**. The T cells in the thymus of each group were concatenated after descending to a unified standard by flowsom dimensionality reduction analysis, and then divided into four subgroups. The blue represents the CD8 (+)CD4 (−) T subgroup; green is the CD4 (+)CD8 (−) T subgroups; orange is the DP T subgroups; and red is the DN T subgroups. **(A, b–e)**. The t-sne dimension reduction graph of each experimental group, GBT, GBT-S, GBT-C, GBT-D in turn. **(B)** Manual analysis of T cell differentiation **(a)** and proliferation **(b)** in the thymus. From left to right are GBT, GBT-S, GBT-C, GBT-D. **(C, D)** are statistical graphs of **(B, a, b)**. **(E)** The PI of thymic T cells of experimental groups. The data are expressed as the mean ± SEM (n = 6). *P < 0.05, **P < 0.01 vs. GBT. ^#^ *P* < 0.05, ^##^ *P* < 0.01 vs. GBT-S. ^▲^ *P* < 0.05 vs. GBT-C.

Similar to the method of T cell analysis in the marrow, we preliminary analysed the distribution of T cell subsets in the thymus. The t-sne results of T cells in the thymus of experimental groups are shown in Figure 13A(a–e). The proportion of CD4(+)CD8(−) T cells was the highest in the thymus of MA rats, followed by CD8(+)CD4(−) T cells, DP, and DN.

Then, the t-sne analysis results were further verified, and we found that only the GBT’s dominant subset was DP, and the other groups were CD4(+)CD8(-)T cell (Figure 13B(a)). In the GBT, the CD4(+)CD8(−) T and CD8(+)CD4(−) T cells were decreased, and DP was increased (Figures 13B(a), C). Then, we further analysed the proliferation of T cell subsets in the thymus, showing that the G1 phase was decreased and S and G2-M phases were increased in GBT (Figures 13B(b), D). Meanwhile, the PI also increased (Figure 13E).

In the other groups, GBT-C could reduce the proportion of S phase (Figures 13B(b), D), and the GBT-D could reduce the proportion of G1 (Figures 13B(b), D) and increase the proportion of G2/M phase (Figures 13B(b), D), but there was no significant difference in the proportion of T cell subsets (Figures 13B(a), C) and the other cell cycle phases (Figures 13B(b), D) in thymus.

In conclusion, GBD containing kansui-liquorice could maintain the dominant cell subsets in the thymus of MA rats and promote the proliferation of thymocytes.

#### 3.3.3 Effects of GBD with or without kansui-liquorice on NPs-NPRs-cGMP-PKGⅡ pathway in MA rats

According to the network pharmacology predicted results, there was not enough evidence to prove the correlation between GBD and fluid metabolism; only the preliminary KEGG analysis (Figure 3) showed that RAAS and cGMP-PKGⅡ pathways are related to GBD. However, GBD has a definite clinical water-expelling effect. GBD is related to RAAS (Liu, 2014); NPs (with signals transmitted through the cGMP-PKGⅡ pathway) and RAAS systems are mutually antagonistic. Therefore, we are interested in the different effects of GBD with or without kansui-liquorice on the NPs system.

ANP and BNP, collectively called NPs (Potter, 2011), bind to NPR-A receptors to activate cGMP/PKGⅡ signalling, which exerts a natriuretic and hydrophilic effect (Brignone et al., 2021). In this study, the contents of ANP and BNP in circulating blood, the gene expression of ANP, BNP, NPR-A, and PKGⅡ, and the protein content of cGMP in the kidney was detected to explore the effect of GBD with or without kansui-liquorice on the NPs-NPRs-cGMP-PKGⅡ pathway in MA rats.

##### 3.3.3.1 Effects of GBD with or without kansui-liquorice on the levels of NPs in the peripheral blood of MA rats

**FIGURE 14:**
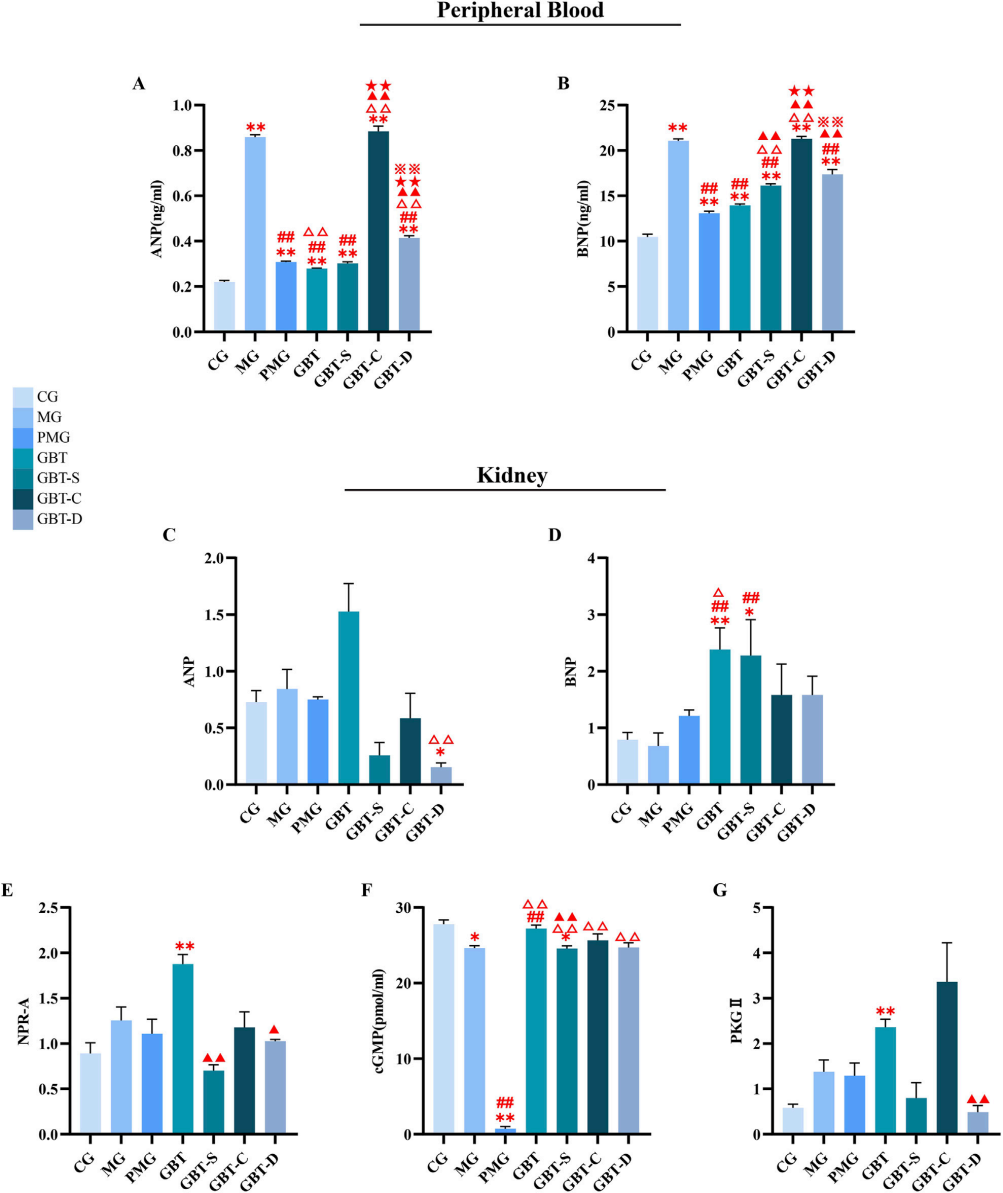
Effect of GBD with or without kansui-liquorice on NPs-NPRs-cGMP-PKGⅡ pathway. The levels of ANP **(A)** and BNP **(B)** in peripheral circulation blood. The gene expression of ANP **(C)**, BNP **(D)**, NPR-A **(E)**, PKGⅡ **(G)**, and protein content of cGMP **(F)** in the kidney. The data are expressed as the mean ± SEM (n = 10). *P < 0.05, **P < 0.01 vs. NG. ^##^ *P* < 0.01 vs. MG.^△^ *P* < 0.05, ^△△^ *P* < 0.01 vs. PMG. ^▲▲^ *P* < 0.01 vs. GBT. ^★★^ *P* < 0.01 vs. GBT-S. ^※※^ *P* < 0.01 vs. GBT-C.

In this study, we determined the content of ANP and BNP in peripheral blood by ELISA, and as shown in Figures 14A, B, the content of ANP and BNP was significantly increased in the MG, whereas they decreased significantly in the administration groups. Among them, the GBT had the lowest NPs level.

In the other groups, they all decreased the content of ANP and BNP when compared with the MG except GBT-C (Figures 14A, B). Among them, PMG, GBT-S and GBT-D decreased gradually (Figures 14A, B), but there was no significant difference among the three groups.

##### 3.3.3.2 Effect of GBD with or without kansui-liquorice on the expression of nps-NPRs-cGMP-PKGⅡ pathway of MA rats

In this study, we further examined the expression of ANP, BNP, NPR-A, PKGⅡ, and cGMP in the kidney. As shown in Figures 14C–G, in the MG, the expressions of related genes and proteins had no significant change compared with the CG. There were also no significant differences between the administration groups (Figures 14C–G). Whereas, in the GBT, the above indicators were increased, which indicated that GBT could activate the NPs/NPRs/cGMP/PKGⅡ pathway to a certain extent.

In summary, GBD reduced the levels of ANP and BNP in the circulating blood, and activated the NPs/NPRs/cGMP/PKGⅡ pathway to some extent effect in the kidney.

## 4 Discussion

According to traditional Chinese medicine theory, kansui is considered incompatible with liquorice. However, there is no consensus in the academic community. The classic formula “Gansui Banxia decoction (GBD)” (the composition of GBD includes kansui and liquorice) can promote the discharge of abnormally accumulated fluids in the body and is primarily used in treating cirrhosis ascites, pleural fluid, and cancerous ascites (Liu et al., 2014). Therefore, this study used GBD as a carrier to explore the role and mechanism of kansui-liquorice in the formulation under the pathological conditions (malignant ascites).

### 4.1 Pharmacokinetics and network pharmacology of GBD with or without kansui-liquorice

In this study, we first investigated the metabolic differences of the important components of GBD with or without kansui and liquorice in MA rats.

The chemical components of chinese materia medica are complex and limited by the current technical means, we selected the most typical chemical components for drug metabolism study.

In modern research, triterpene saponins and flavonoids are the most common compounds in liquorice, which are also the most important bioactive components of liquorice. Among them, the representative component of triterpene saponins is glycyrrhizic acid, and the representative component of flavonoids is liquiritin (Shang et al., 2022). Glycyrrhetinic acid is the main active metabolite of glycyrrhizic acid (Roohbakhsh et al., 2016). The main toxic-active components of kansui are kansuinine A and kansuinine B (Jing et al., 2015). However, kansuinine B did not achieve the desired detection effect in preliminary experimental tests, which may be related to our method of administering kansui in powdered form. Therefore, only the kansuinine A was analyzed. The active components of radix paeoniae alba ranked first in the component-target analysis of network pharmacology, and the main active component of radix paeoniae alba is paeoniflorin. Thus, in this experiment, we first observed the differences in the pharmacokinetics of five important chemical components-glycyrrhizic acid, liquiritin, paeoniflorin, glycyrrhetinic acid, and kansuinine A-in MA rats when kansui and liquorice were included or excluded from GBD.

According to the pharmacokinetic results, kansui reduced the Cmax of glycyrrhizic acid and decreased its bioavailability to some extent, but the Cmax and bioavailability of glycyrrhetinic acid significantly increased (Figure 1; Table 3).

Glycyrrhetinic acid is the main metabolite of glycyrrhizic acid, and both have similar pharmacological effects and a wide range of pharmacological effects, such as anti-inflammatory, immunomodulatory, and anti-tumor effects (Shinu et al., 2023; Bordbar et al., 2012).

However, glycyrrhizic acid can be rapidly metabolized to glycyrrhetinic acid by oral administration (Feng et al., 2015). Glycyrrhetinic acid, but not glycyrrhizic acid, has been found to inhibit the proliferation of triple-negative breast cancer cell line MDA-MB-231 *in vitro*, and can be combined with etoposide to exert a synergistic effect (Cai, 2017)Thus, glycyrrhetinic acid is thought to be responsible for most of the pharmacological effects of glycyrrhizic acid.

Thus, we speculated that kansui could promote the metabolism of glycyrrhizin acid to glycyrrhetinic acid, thereby enhancing the bioavailability of liquorice to get the best out of liquorice. Therefore, one of the reasons for the synergistic effect of kansui and liquorice may be related to the increased bioavailability of glycyrrhetinic acid.

The chemical site GS-3 of Glycyrrhiza glabra significantly promoted the proliferation of splenic lymphocytes, proving that the GS-3 site is an inflammatory toxicity site group of kansui. A total of 8 compounds were isolated from this site, and kansuinine A was one of them (Zhang et al., 2009). We found that liquorice could reduce reduce the leaching of kansuinine A (Figure 1; Table 3), thereby decreasing its potential to cause inflammatory damage. Therefore, the reason for the synergistic effect of kansui and liquorice when used together may also be related to its reduction of the leaching of the toxic substance-kansuinine A-from kansui.

In conclusion, from the perspective of pharmacokinetics, the reason for the enhanced efficacy when using kansui and liquorice together may be related to the promotion of liquorice metabolism and the reduction of the leaching of toxic substances of kansui, which explains the rationality of GBD to a certain extent, and provides material basis data support for the “synergistic effect” of kansui-liquorice combination.

Then we further used network pharmacology to predict the target and mechanism of GBD. We conducted GO and KEGG analyses on the intersected genes obtained by intersecting the drug targets of GBD with the targets of MA disease. In terms of GO BP, GBD may be related to the differentiation of immune cells (Figure 3). In the KEGG pathways, GBD is associated with cancer pathways, tumor-related immune cells (such as T and NK cells), inflammation-related pathways, fluid metabolism, and the cell cycles (Figure 3). Later, using the MCODE algorithm from the Metascape database, the intersected genes were classified into nine key functional modules (Figures 4, 5; Table 4, 5). It was found that the functions of the intersected genes were mainly concentrated on transmembrane cell motility, protein phosphorylation, cell migration, cell cycle regulation, hormone synthesis and metabolism, inflammatory response and Ga2+ transmembrane motility (Table 5). By screening the core KEGG pathways through the KEGG database, we found that the most important pathways are mainly concentrated in immunity, inflammation, and the cell cycle (Figure 6; Table 6, 7). Afterwards, the GeneMANIA software was used to predict the hub-genes within the intersected genes (Supplementary Figure 5), revealing that the hub-genes are related to immune inflammatory responses and cell cycle regulation (Figure 7B). Based on the predictive results of network pharmacology, we found that GBD plays an important role in anti-tumor and regulating immunity and inflammation, with T cells and NK cells being mentioned.

In the pharmacokinetic analysis, it was found that the combination of kansui with liquorice could increase the level of glycyrrhetinic acid. Combined with network pharmacology, we further analyzed the targets and pathways regulated by glycyrrhetinic acid. The research methods were similar to those in 2.2 Network Pharmacology in Materials and Methods, as detailed in Supplementary Material 2 *(Part 3: Prediction of the mechanism of glycyrrhetinic acid)*.

Compared with the KEGG and GO BP enrichment results of glycyrrhetinic acid and GBD, we found that GBD basically covered the enriched results by glycyrrhetinic acid. However, in the KEGG enrichment results, glycyrrhetinic acid were more inclined to inflammatory, immune and anti-tumor related pathways. And in the GO BP enrichment results, the anti-inflammatory and immune-related cell functions became more prominent (Supplementary Figures 9A, B). Meanwhile, when the core genes of glycyrrhetinic acid were analyzed, their functions were also related to immune-inflammatory response (Supplementary Figure 9D). The above results further verified that the combination of kansui and liquorice may enhance the efficacy by regulating the immune response.

The components of traditional Chinese herbs are complex, and limited by the current research methods, we study the drug composition is limited. So more differential components need to be excavated to evaluate their influence on the pathological state of MA.

### 4.2 Effects of GBD with or without kansui-liquorice on the immune system in MA rats

To resist the invasion by pathogens, humans have evolved a powerful immune system, including the innate and adaptive immune systems. The innate immune system can respond rapidly, but adaptive immunity can produce more powerful and effective immune response (Parkin and Cohen, 2001). Based on the results of the network pharmacology, we selected intrinsic immune cells-NK cells and adaptive immune cells-T cells to further explore the effects of GBD with or without kansui-liquorice on cellular immunity.

NK [CD3 (−)CD161 (+)] cells are an important part of innate immunity and have cytotoxic effector functions. NK cells play an important role in eliminating malignant cells and limiting tumor metastasis (Liu et al., 2021). NKT [CD3 (+)CD161 (+)] cells are also an important part of the innate immune system, but belong to a particular T cell subset, which can express specific antigens on the surface of both T and NK cells. Similar to NK cells, NKT cells have potent anti-tumor activity (Liu et al., 2021). Consistent with previous results (Huo et al., 2024), we also found that NK cells were dominant in the PB and TME of MA rats among groups (Figures 8–11; Tables 8, 9), especially in the TME. Therefore, we hypothesized that NK cells are important for anti-tumor immunity in MA rats. However, GBD containing kansui-liquorice had no noticeable effect on NK or NKT cell populations compared with removing it (Figures 9B, 11C).

T cells are an important cell population in adaptive immunity and play an important role in controlling tumorigenesis and development. Although GBD had no special effect on NK cells in the MA rats, it could increase the percentage of T cells (Figures 8–11). The simplest distinction between T cells is CD4 (+) or CD8 (+) T cell subsets. The presence of tumor-infiltrating lymphocytes, especially CD8 (+) T cells, is a positive prognostic marker for a variety of solid tumors and is a key factor in anti-tumor activity (Thommen and Schumacher, 2018). CD28 is a common stimulus molecule for CD8, providing the activation signal that prompts CD8 (+) T cell activation to exert anti-tumor effects (Esensten et al., 2016). According to previous studies, CD8 (+)CD28 (+) T cells in the TME of MA rats were significantly reduced, and GBD could significantly increase it (Huo et al., 2024). In this experiment, we further found that the addition of kansui-liquorice to the GBD had a more significant effect in promoting the increase of CD8 (+)CD28 (+) T cells both in the PB and TME of MA rats (Figure 9A(d), D; Figures 11B, C). In the TME, malignant cells induce immune cell exhaustion and anergy, resulting in a decrease of cytotoxic immune cells and an increase of immunosuppressive cells. Therefore, malignant cells undergo immune escape, leading to the inability of the body to produce effective anti-tumor immunity (Crespo et al., 2013; Thommen and Schumacher, 2018). Regulatory T cells (Tregs), characterised by the expression of the master transcription factor forkhead box P3 (Foxp3), suppress anti-tumor immunity and obstruct effective anti-tumor immune responses in tumor-bearing hosts (Li et al., 2020). GBD can effectively reduce Tregs cells in the PB [CD4 (+)CD25 (+) Foxp3 (+)] and TME [CD8 (+)CD25 (+)Foxp3 (+)] cells of MA rats (Huo et al., 2024). In this experiment, we further compared GBD with or without kansui-liquorice, and found that the GBD with kansui-liquorice was effective in reducing the immunosuppressive cells in the PB [CD4 (+)CD25 (+)Foxp3 (+)] and TME [CD8 (+)CD25 (+)Foxp3 (+)] cells (Figures 9A(e), D; Figures 11A(c), C).

In this study, by comparing the enrichment analysis of hub genes between glycyrrhetinic acid and GBD, we found that glycyrrhetinic acid may be more likely to target the functions of immune regulation. Glycyrrhetinic acid has been reported to have a wide range of immunomodulatory effects. It has been found that glycyrrhetinic acid could stimulate the maturation and function of dendritic cells as antigen presenting cells, thereby initiating primary T cell immune responses (Bordbar et al., 2014). Other studies have shown that glycyrrhetinic acid regulates T cell proliferation and activation by blocking Kv1.3 potassium channel (Fu et al., 2013). Further experiments showed that the combination of kansui and liquorice may enhance the effect by regulating the function of immune cells, including increasing the number of effector T cells and decreasing the number of immunosuppressive cells. Combined with the above results, we speculated that the synergistic effect of liquorice and kansui may be related to the increased content of glycyrrhetinic acid, which is related to the targeted regulation of T cells.

In summary, regardless of whether GBD contained kansui-liquorice, there was no significant effect on NK and NKT cells in MA rats. However, GBD containing kansui-liquorice could increase CD8 (+)CD28 (+) T cells in the PB and TME and reduce Treg cells in the PB [CD4 (+)CD25 (+)Foxp3 (+)] and TME [CD8 (+)CD25 (+)Foxp3 (+)] of MA rats. Therefore, the mechanism of enhanced efficacy of GBD containing kansui-liquorice compared with removing it may be related to its promotion of T cell immunity in MA rats.

### 4.3 Effects of GBD with or without kansui-liquorice on T cell development in MA rats

T cells originate from marrow progenitor cells, which migrate to the thymus for maturation and selection, and then export to the peripheral blood, resulting in marrow progenitor cells lacking CD4 and CD8 co-expressed receptors differentiate into CD4 (+) or CD8 (+) single positive (SP) cells after dual selection, and then are transported to the periphery to perform effective immune functions (Kumar et al., 2018). Therefore, the marrow and thymus are important immune organs of the body. In the marrow, DN T cells were the dominant subset, whereas, in the thymus, DP T cells were dominant (Kumar et al., 2018). The changes of the dominant subset in the thymus and marrow may indicate the changes of their functions. GBD could exert an effective anti-tumor effect by enhancing T cell immunity in MA rats, so we further explored the function of the T cell development sites, which were the marrow and thymus.

In cancer patients, abnormal marrow proliferation can be found, leading to an increase in immature immune cells, which promotes tumor development and metastasis (Pinho and Frenette, 2019). In the early stages of our research, we similarly found that the marrow proliferation was increased in MA rats, and that GBD could inhibit the excessive proliferation to a certain extent (Huo et al., 2024). In this experiment, we found that the proportion of T cell subsets in the marrow of GBD with or without kansui-liquorice was not significantly altered (Figures 12B(a), C), whereas the proliferation of GBD containing kansui-liquorice was decreased (Figures 12B(b), D, E). The thymus is the main site of T cell development and is essential for the production of T cell functional immunity. The size and number of cells in the thymus decrease with age, known as thymic involution or atrophy. This involution leads to impaired T cell development and changes in the composition and function of the T cell pool, which increases the risk of developing cancer (Gulla et al., 2023). Cancer-related thymic involution has also been observed in cancer patients (Li and Záñiga-Pflücker, 2023). In addition to structural atrophy, the degenerated thymus is functionally impaired, including the decreased proportion of DP cells and proliferation of thymocytes (Aw et al., 2009; Aw et al., 2010; Liang et al., 2022). MA rats have cancer-related thymic involution, both structurally and functionally. GBD has a limited effect on reversing the atrophy of the thymus, but it can improve the function to some extent (Huo et al., 2024). In this study, we further analysed the effects of GBD with or without kansui-liquorice on cancer-related thymic involution of MA, and the results showed that there was a significant difference in the proportion of T cell subsets in the thymus among groups, and the proportion of DP T cells was significantly increased in GBD containing kansui-liquorice (Figures 13B(a), C). Beyond that, the addition of kansui and liquorice also could promote the proliferation of the thymus (Figures 13B(b), D, E).

In conclusion, GBD containing kansui-liquorice could effectively inhibit the excessive proliferation of marrow and improve the immune function of the thymic involution, thereby ensuring the output of effective functional T cells in the periphery. Therefore, the effect of GBD containing kansui-liquorice on enhancing T cell immunity may be related to improving the function of the marrow and thymus.

### 4.4 Effects of GBD with or without kansui-liquorice on NPs-NPRs-cGMP-PKGⅡ pathways in MA rats

Although network pharmacology results did not provide enough evidence to prove the correlation between GBD and fluid metabolism, GBD has a definite clinical water-expelling effect. Moreover, one of the arguments supporting the combined use of kansui and liquorice is that their combination enhances the water-expelling effect of kansui. Therefore, it is necessary for us to conduct an in-depth study on the impact of GBD with or without kansui and liquorice on the fluid metabolism of MA rats.

In MA disease, the increased ascites lead to insufficient peripheral circulating blood volume when the renin angiotensin aldosterone system (RAAS) is activated, eventually leading to vasoconstriction, increased aldosterone secretion, water and sodium retention, and other biological effects, all of which further worsen the ascites disease (Becker et al., 2006; Cavazzoni et al., 2013; Garrison et al., 1987). Therefore, the activation of the RAAS system is an important factor in the aggravation of ascites. According to previous experimental results, GBD is closely related to the RAAS (Liu, 2014), which can alleviate the activation of RAAS caused by MA. Natriuretic peptides A and B (ANP and BNP) are polypeptide hormones that antagonizing RAAS system with powerful diuretic, natriuretic, vasodilation and inhibition of renin and aldosterone secretion, and play an important role in systemic and local regulation by binding to natriuretic peptide receptors (NPR-A, NPR-B, NPR-C) in target organs (Brignone et al., 2021; Laragh, 1985; Maack et al., 1984; Theilig and Wu, 2015 Volpe, 2014; Volpe et al., 2016). The kidneys, as one of the most important target organs for NPs, express genes and proteins for three types of NPs and their receptors, forming a local renal natriuretic peptide system (Lin and Hong, 2011). Therefore, this study further explores the effects of GBD with or without kansui and liquorice on the renal natriuretic peptide system.

ANP and BNP are closely related to the water-sodium metabolism. Firstly, the levels of ANP and BNP in peripheral blood were detected. The results showed that GBD did not significantly increase the level of ANP and BNP of MA rats, which may be related to the MA rats were in the terminal stage of the disease. The levels of ANP and BNP in peripheral circulation were positively correlated with the level of heart failure (Mangiafico et al., 2013). With the progression of the MA disease, the RAAS system continues to be activated, and the secretion of ANP and BNP increases. The high level of NPs is not enough to counteract the biological effects of RAAS over-activation, which is called natriuretic peptide resistance (Dong et al., 2017; Gopi et al., 2013; Mangiafico et al., 2013; Potter, 2011). Therefore, the levels of ANP and BNP in the peripheral blood at this time reflected the volume of circulation in MA rats. In this experiment, we found that MA rats had high levels of NPs in the peripheral blood, and GBD with or without kansui-liquorice could reduce its level, among them, GBD with kansui and liquorice had the lowest degree (Figures 14A, B), which consistented with the previous findings that GBD reduced the ascites formation in MA rats (Huo et al., 2024). The above results indirectly confirm that GBD containing kansui and liquorice could reduce the abnormal fluid accumulation in the body. At the same time, to eliminate the influence of cardiac secretion of NPs, we further examined the mRNA expression of ANP and BNP in the kidneys. The results showed that GBT group had highest mRNA expression, and the difference in BNP was particularly significant.

ANP and BNP mediate biological effects by activating cGMP-PKGⅡ through binding to the NPR-A receptor (Lin and Hong, 2011). Over-activated RAAS mediated the high levels of AngⅡ, which could downregulate the binding pathway between ANP and NPR-A (Gopi et al., 2013), abnormal signal transduction of NPs, and increase degradation of cGMP (Potter, 2011). According to the expression of NPR-A and cGMP-PKGⅡ pathway in the kidney, there was no significant difference between the groups in MA rats (Figures 14C–G). However, GBT had higher expression (Figures 14C–G), indicating that GBD containing kansui-liquorice could increase the expression of the NPs-NPRs-cGMP-PKGⅡ pathway to a certain extent.

Studies conducted over the past 20 years support a relevant role of NPs in a complex network connecting the cardiovascular and immune systems, with researchers suggesting that locally released NPs can act in a cytokine-like manner to exert anti-inflammatory and anti-tumor effects (Paolo, 2014). This study found that the combination of kansui and liquorice has effects on activating NPs/NPR-A/cGMP/PKGⅡ pathway and regulating immunity, but the specific mechanism needs to be further studied.

In conclusion, to a certain extent, GBD can activate the NPs-NPRs-cGMP-PKGⅡ pathway to antagonize the activation of RAAS caused by MA, and thus improve the severe peripheral circulation disorder in MA rats, and the combination of kansua and liyuorice can enhance the above biological effects.

### 4.5 The controversy on the incompatibility of kansui and liquorice

Since the Jin dynasty, Zhang Zihe named the summarized contraindications of Chinese herbal medicine “eighteen antagonisms,” and this term has been used until now. However, there has never been a consensus among physicians on whether the “eighteen antagonisms” are absolute contraindications. Kansui-liquorice is one of the “eighteen antagonisms,” and modern research suggests that their “antagonism” includes two aspects: increased toxicity and reduced efficacy. In terms of increased toxicity, numerous pharmacological experiments have verified that the co-administration of kansui and liquorice increases the precipitation of toxic substances, such as euphadienol (Duan et al., 2018). Additionally, glycyrrhizic acid can form molecular complexes with the steroidal-terpenoid compounds in kansui, promoting the dissociation of these steroidal-terpenoid components (Chen, 1984). In addition, kansui and liquorice jointly act on the metabolism hepatic enzymes with detoxification functions, exerting inducing or inhibiting effects, causing the accumulation of toxic components or converting them into new toxic components, resulting in an increase in toxicity (Dai et al., 2005a; Dai et al., 2005b). Preclinical experiments have also confirmed that the combination of kansui and liquorice has certain toxic side effects on the heart, liver, kidneys, and intestinal flora balance of experimental animals (Yu, 2018; Zhou et al., 2023). In terms of reduced efficacy, kansui and liquorice have opposite effects. Kansui has water-expelling properties. Research has found that kansui may exert its natriuretic and diuretic effects by inhibiting the secretion of renin and enkephalinase or reducing their activity. On the other hand, liquorice could lead to the accumulation of cortisol, acting like a mineralocorticoid, which results in water and sodium retention (Calò et al., 2004; Liu and Fan, 2015). Therefore, liquorice and kansui have opposite effects on regulating water and electrolyte excretion.

However, both liquorice and kansui are associated with various anti-tumor, anti-inflammatory, and immune regulation pathways (Liu and Fan, 2015), and multiple preclinical studies have verified that their combined use can promote anti-inflammatory, immune regulation, and inhibition of malignant tumors (Huo et al., 2024; Pastorino et al., 2018; Shen et al., 2016a). In this experiment, we also demonstrated that under formula conditions, kansui and liquorice can exert dual effects of regulating immunity and promoting fluid metabolism, promoting the resolution of malignant ascites. Therefore, we believe that the combined use of kansui and liquorice is not an absolute contraindication. Although studies have shown that co-administration can lead to the leaching of toxic substances, this experiment found that under the formulation conditions, liquorice could reduce the dissolution of the toxic substance -kansuinin A-from kansui, while promoting the metabolism of glycyrrhizinic acid to glycyrrhizinic acid, thereby enhancing the bioavailability of licorice. However, limited by current analytical techniques, no method can accurately describe all possible compounds within the sample, so it is still impossible to determine the specific changes in the toxicity-efficacy chemical compositions after the combined use of kansui and liquorice. At the same time, a large number of preclinical studies have found that under pathological conditions, kansui and liquorice do not produce significant organ toxicity in a formulated environment (Liu, 2014; Lin, 2016; Wang et al., 2016), which is consistent with our previous experimental results. Interestingly, there is a dose-toxicity/efficacy inflection point for the combination of kansui and liquorice. Increasing the proportion of kansui reduces toxicity but decreases the water-expelling effect; increasing the proportion of liquorice increases the water-expelling effect but enhances toxicity (He et al., 2018; Liu, 2014; Shen et al., 2015; Shen et al., 2016b). Additionally, vinegar processing of kansui can reduce its toxicity, but the water-expelling effect is also relatively reduced (Cao et al., 2011; Liu et al., 2020). Therefore, the differences in formulation conditions, pathological environment, dosage, and preparation methods all have a certain impact on the toxicity-efficacy effects after the combined use of kasnui and liquorice. Thus, based on the results of this experiment, we believe that the combined use of kansui and liquorice is not an absolute compatibility contraindication. Their combined use can exert effective immune regulation and promote fluid metabolism, providing theoretical support for the clinical use.

In summary, according to the results of this experiment, under the guidance of the TCM theory of “pattern identification/syndrome differentiation and treatment,” kasnui and liquorice can be used together with other herbs to alleviate diseases related to abnormal fluid accumulation in the body, such as malignant ascites. But, how to use kansui and liquorice appropriately in clinical practice, and how to seek a balance between toxicity and efficacy, are issues that we still need to address further.

The “eighteen antagonisms” is the core content of prohibited combination in TCM prescriptions, and are also an important aspect of the rational use of chinese medicinal, equivalent to the adverse reactions, contraindications, and precautions in the instructions. They also serve as a crucial reference for TCM practitioners in clinical practice. *The Chinese Pharmacopoeia* has included the contents of “eighteen antagonisms” in the “Attention” of chinese medicinal. Due to the contraindication of “eighteen antagonisms,” the combination of kansui and liquorice is limited in clinical practice, and its medicinal value has not been fully developed. However, with the continuous enrichment and development of modern medical technology and traditional Chinese medicine knowledge, the “eighteen antagonisms” should continue to evolve and update. According to the results of this study, we believe that kansui and liquorice are not absolutely incompatible, and the combination of kansui and liquorice could play a “water-expelling” effect by regulating the T cell immune function and activating the “NPs/NPR-A/cGMP/PKGⅡ” pathway, which provides more possibilities for the treatment of ascites, and provides an important experimental basis for the clinical application of “eighteen antagonisms,” so as to guide the clinical rational use of “eighteen antagonisms”. As a contemporary Chinese medicine practitioner, we should keep the right and innovate, not only to inherit the culture of TCM, but also to break the limitations of our thoughts and to try and practice continuously, so that we can clarify the application value of “eighteen antagonisms”.

## 5 Conclusion

In this study, we first predicted that GBD might exert an effective “water-expelling” medicinal effect by enhancing cellular immunity through network pharmacology. Then we provided material evidence for the rationality of the combined application of kansui-liquorice in GBD through pharmacokinetics. Combining the predictions of network pharmacology and the results of a previous study, we investigated the different effects of GBD with and without kansui-liquorice in the treatment of MA on the NPs system and cellular immunity, and found that GBD containing kansui-liquorice was superior to the experimental group without kansui-liquorice in promoting the activation of the NP signalling pathway and enhancing T cell immunity. Therefore, we believe that kansui and liquorice are important herbs of GBD, and their combined application enhances the direct (activation of the NPs/NPRs/cGMP/PKGⅡ pathway) and indirect (enhancement of T cell immunity) water-expelling effects of GBD, to promote the clinical remission of MA. However, how to use kansui and liquorice appropriately in clinical practice, and how to seek a balance between toxicity and efficacy, are issues that we still need to address further.

## Data Availability

The original contributions presented in the study are included in the article/Supplementary Material, further inquiries can be directed to the corresponding authors.

## Glossary

### Symbol Description

ANOVA: Analysis of variance
ANP: Atrial natriuretic peptide
BNP: Brain natriuretic peptide
BP: Biological Processes
CC: Cellular Components
CG: Control group
CNKI: China National Knowledge Infrastructure
DEG: Differentially expressed genes
DN: Double negative
DP: Double positive
ELISA: Enzyme-linked immunosorbent assay
ETCM: Encyclopedia of Traditional Chinese Medicine
GBD: Gansui Banxia decoction
GBD-C: Gansui Banxia decoction remove liquorice
GBD-D: Gansui Banxia decoction remove kansui and liquorice
GBD-S: Gansui Banxia decoction remove kansui
GBT: Gansui Banxia decoction group
GBT-C: Gansui Banxia decoction remove liquorice group
GBT-D: Gansui Banxia decoction remove kansui and liquorice group
GBT-S: Gansui Banxia decoction remove kansui group
GEO: Gene Expression Omnibus
GO: Gene Ontology
KEGG: Kyoto Encyclopedia of Genes and Genomes
MA: Malignant ascites
MCODE: Molecular Complex Detection
MF: Molecular Functions
MFI: Median fluorescence intensity
MG: Model group
MRM: Multi Reaction Monitoring
NF-κB: Nuclear factor kappa B
NK: Natural killer
NP: Natriuretic peptide
PB: Peripheral blood
PI: Proliferation index
PMG: Positive medicine group
PPI: Protein interaction
RAAS: Renin angiotensin aldosterone system
SP: Single positive
TCM: Traditional Chinese medicine
TCMSP: Traditional Chinese Medicine Systems Pharmacology
TNF: Tumor necrosis factor
Treg: Regulatory T cell
TME: Tumor micro-environment
TTD: Therapeutic Target Database.

### Abbreviations for each Chinese medicine in network pharmacology

BS: Radix paeoniae alba(Chinese name:白芍, Baishao)
BX: Pinellia ternata(Chinese name:半夏, Banxia)
FM: Honey(Chinese name:蜂蜜, Fengmi)
GC: Liquorice(Chinese name:甘草, Gancao)
GS: Kansui(Chinese name:甘遂, Gansui).

### Abbreviations for each component in pharmacokinetics

6-HF: 6-Hydroxyflavone
BFSQ: Bendroflumethiazide(苄氟噻嗪)
GCCS: Glycyrrhetinic acid(甘草次酸)
GCG: Liquiritin(甘草苷)
GCS: Glycyrrhizic acid(甘草酸)
GSTZ-A: Kansuinine A(甘遂萜脂A)
JBHDN: Tolbutamide(甲苯磺丁脲)
SYG: Paeoniflorin(芍药苷)
AUC: Area under the curve
Cmax: Peak Concentration
T1/2: Half-life time
Tmax: Peak time
