# Supplementary material for: Kansui-liquorice enhances the “water-expelling” effect of Gansui Banxia decoction in rats with malignant ascites by targeting the NPs/NPRs/cGMP/PKGⅡ pathway and T cell immunity
Source: Front Pharmacol. 2025 Apr 28;16:1557717. doi: 10.3389/fphar.2025.1557717 (PMC12067993; doi:10.3389/fphar.2025.1557717)
Supplement: Supplementary file 1 [file DataSheet1.pdf]

Original Research Article

Supplementary Material

Kansui-liquorice enhances the “water-expelling” effect of Gansui Banxia decoction in rats with malignant ascites by targeting the NPs/NPRs/cGMP/PKGII pathway and T cell immunity.

Min Huo[1], Haiyan Liu[1*], Shaohong Chen[1], Lingling Xiu[1], Xue Yu[1], Gansheng Zhong[1*]

Affiliations

[1]School of Traditional Chinese Medicine, Beijing University of Chinese Medicine, Beijing, China

***Correspondence**

Corresponding author 1：Haiyan Liu
E-mail addresses: 700549@bucm.edu.cn
Corresponding author 2：Gansheng Zhong
E-mail addresses: zhonggansheng@sohu.com

This document contains two parts, the Part 1: Supplementary figures; the Part 2: Supplementary tables; the Part 3: Prediction of the mechanism of glycyrrhetinic acid.

Part 1: Supplementary figures

Supplementary Figure 1. The chemical structures of glycyrrhizic acid **(A)**, liquiritin **(B)**, paeoniflorin **(C)**, glycyrrhetinic acid **(D)**, kansuinine A **(E)**, 6-HF **(F)**, and bendrofluazide **(G)**.

A

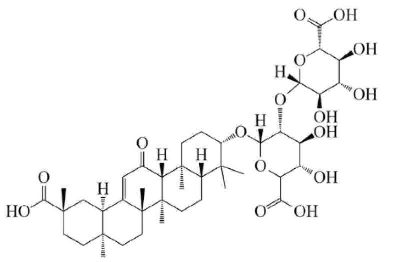

Glycyrrhizic acid

B

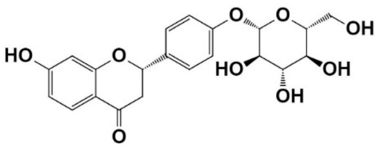

Liquiritin

C

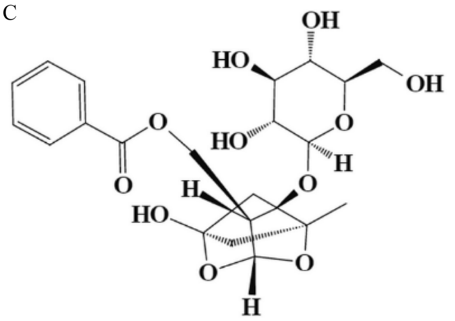

Paeoniflorin

D

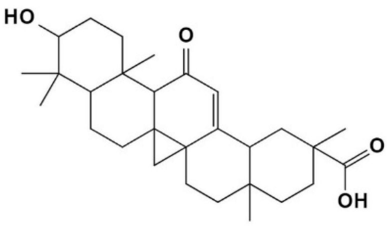

Glycyrrhetinic acid

E

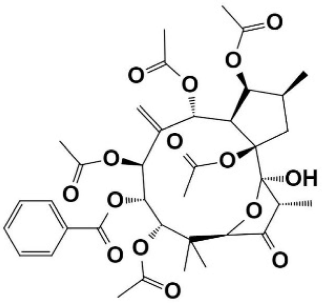

Kansuinine A

F

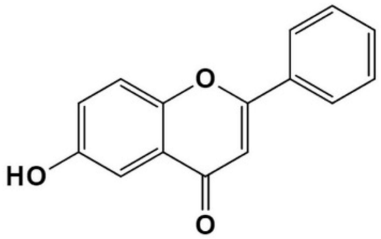

6-Hydroxyflavone

G

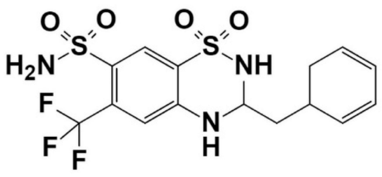

Bendrofluazide

Supplementary Figure 2. Each component of MRM. Glycyrrhizic acid, liquiritin, glycyrrhetinic acid, paeoniflorin, kansuinine A, 6-HF and bendrofluazide (from top to bottom) in blank plasma **(A)**, blank plasma added reference and internal standard solution **(B)**, and plasma after administration **(C)**.

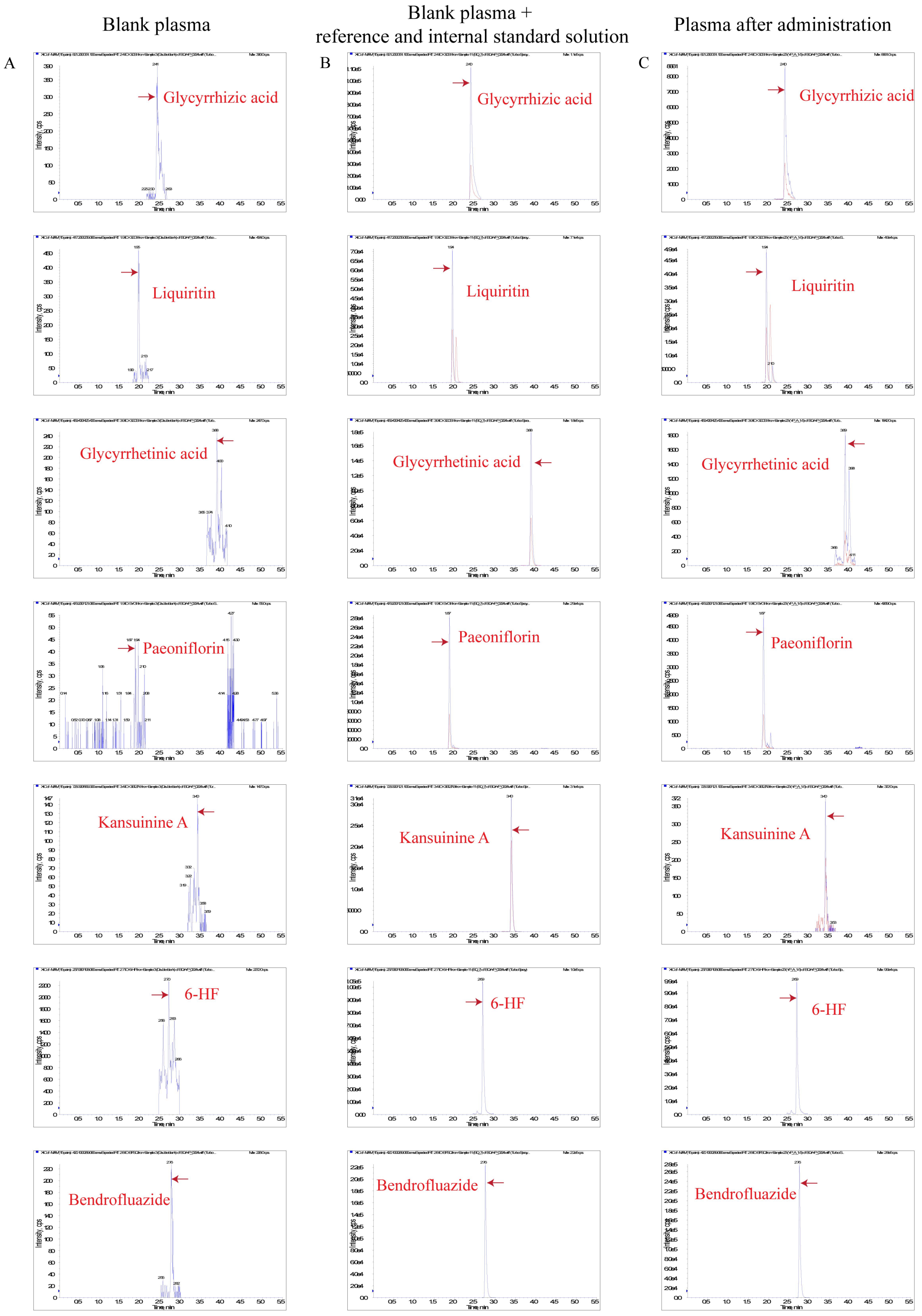

Supplementary Figure 3. The volcano plot presents the differentially expressed genes (DGEs) screened from the GSE15831 **(A)**, GSE39204 **(B)**, and GSE73168 **(C)**. The blue dots represent the down-regulated genes in the model control group, the red dots represent the up-regulated genes, and the gray dots represent the genes that did not show significant difference. The dots were higher, the greater the possibility of difference from the blank control group. The dots were farther from the middle line, the greater the gap from the blank control group.

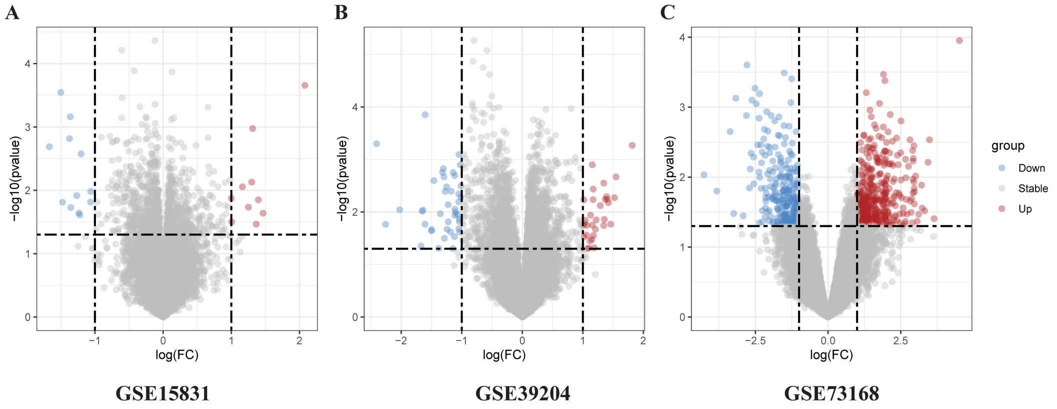

Supplementary Figure 4. The intersection targets of potential drug and MA-related targets. The blue circles represent 1,866 MA-related targets, and the pink circles represent 1,166 potential drug therapeutic targets of GBD. There were 196 intersection targets between them.

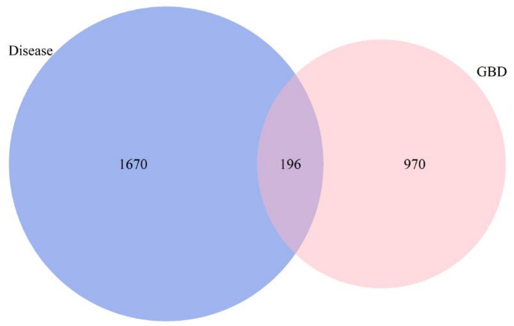

Supplementary Figure 5. Venn diagram of hub genes. Nine hub genes were obtained after intersection of the hub genes screened under different algorithms.

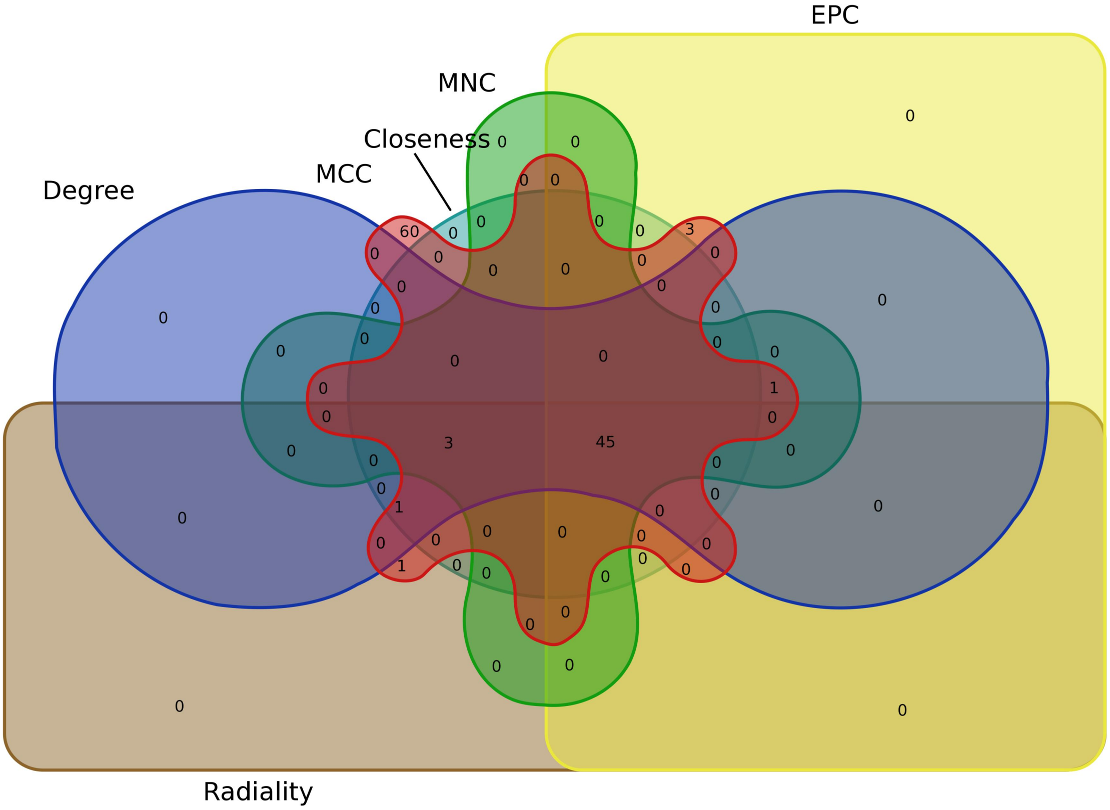

Supplementary Figure 6. The flow cytometry gate steps setting of peripheral blood. **(A)** Demonstrate all flow fluorescence signals with FSC-A, SSC-A as X, Y axes and select all target cells. **(B)** With "all target cells" set as a new window, FSC-A and APC-Cy7-A are used as X, Y axis to screen live cells in target cells. **(C)** With "live cells" set as a new window, screen NK cells (top left), NKT cells (top right), CD3 T cells (bottom right) with CD3 and CD161 as X, Y axes. **(D)** With "NKT" cells set as a new window, screen NKT-CD8 (top left), NKT-DP (top right), NKT-CD4 (bottom right) with CD4, CD8 as X, Y axes. **(E)** With "CD3 T" cells set as a new window, screen CD8 T cells (top left), DP (top right), CD4 T (bottom right), and DN (bottom left) with CD4, CD8 as the X, Y axis. **(F)** With "CD8 T" cells set as a new window, screen CD8CD28 T cells (top right) with CD28, CD8 as the X, Y axis (upper right). **(G)** With "CD4 T" cells set as a new window, CD25, Foxp3 was used as the X, Y axis to screen Treg cells (top right).

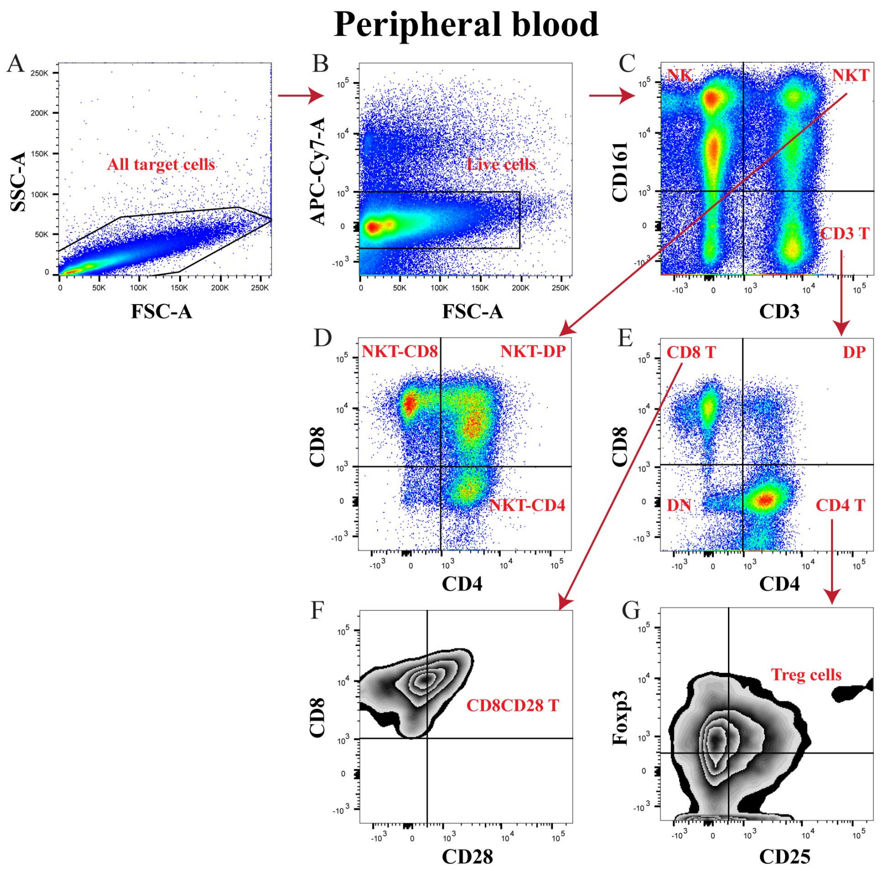

Supplementary Figure 7. The flow cytometry gate steps setting of tumor microenvironment. **(A)** Demonstrate all flow fluorescence signals with FSC-A, SSC-A as X, Y axes and select all target cells. **(B)** With "all target cells" set as a new window, CD45, APC-Cy7-A are used as X, Y axis to screen live cells in target cells. **(C)** With "Live CD45 cells" set as a new window, screen NK cells (top left), CD3 T cells (bottom right) with CD3 and CD161 as X, Y axes. **(D)** With "CD3 T" cells set as a new window, screen CD8 T cells (top left), DP (top right), CD4 T (bottom right), and DN (bottom left) with CD4, CD8 as the X, Y axis. **(E)** With "CD8 T" cells set as a new window, screen CD8CD28 T cells (top right) with CD28, CD8 as the X, Y axis. **(F)** With "CD8 T" cells set as a new window, CD25 and Foxp3 was used as the X, Y axis to screen CD8 Treg cells (top right).

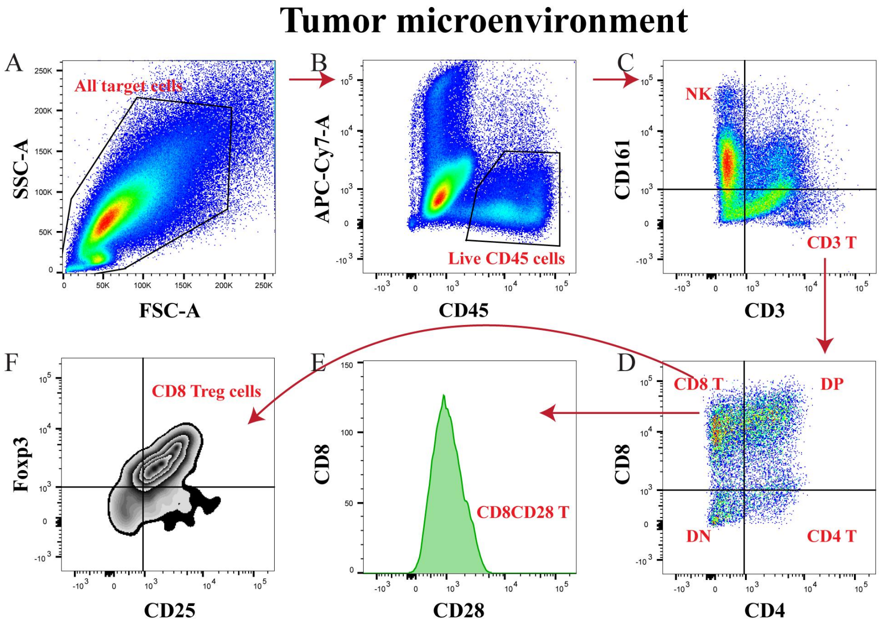

Supplementary Figure 8. The flow cytometry gate steps setting of bone marrow and thymus. **(A)** Demonstrate all flow fluorescence signals with FSC-A, SSC-A as X, Y axes and select all target cells. **(B)** With "all target cells" set as a new window, FSC-A and FSC-W are used as X, Y axis to screen single cells in target cells. **(C)** With "single cells" set as a new window, CD3 and APC-Cy7-A are used as X, Y axis to screen live CD3 T cells in the single cells. **(D)** With "Live CD3 T" cells set as a new window, screen CD8 T cells (top left), DP (top right), CD4 T (bottom right), and DN (bottom left) with CD4, CD8 as the X, Y axis. **(E)** With "Live CD3 T cells" set as a new window, screen different cell cycle (including G0, G1, S and G2~M phase) with 7-AAD, Ki-67 as the X, Y axis.

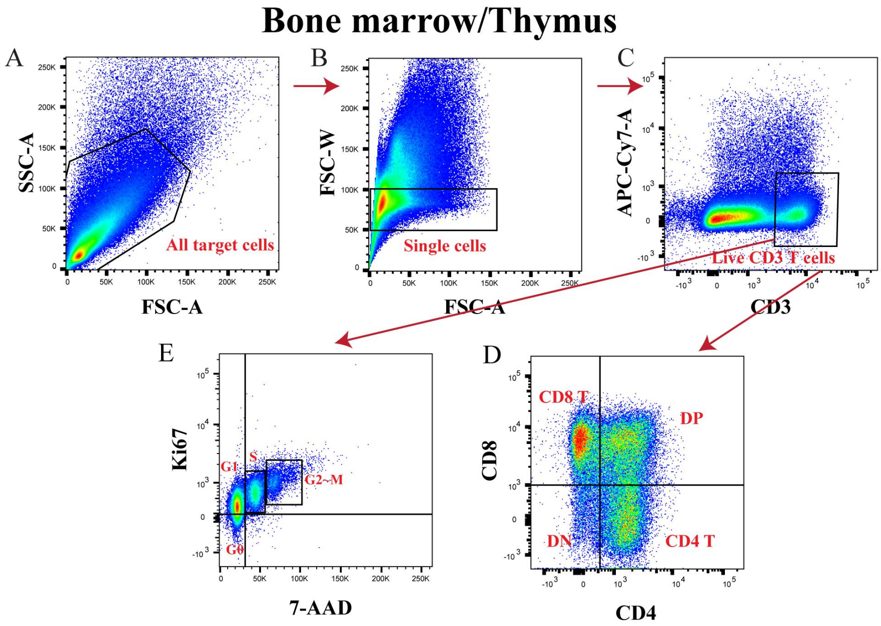

Supplementary Figure 9. Bubble plots of KEGG **(A)** and GO BP **(B)** analysis results for the 27 key therapeutic targets of glycyrrhetinic acid. **C** is PPI network of the 27 key therapeutic targets. **D** is co-expression network of hub genes. The larger the bubble, the more intersection genes are enriched in this item; the smaller the bubble, the fewer intersection genes. The redder the colour and the further away from the X-axis, the smaller the P-value of this entry; the opposite is even bigger.

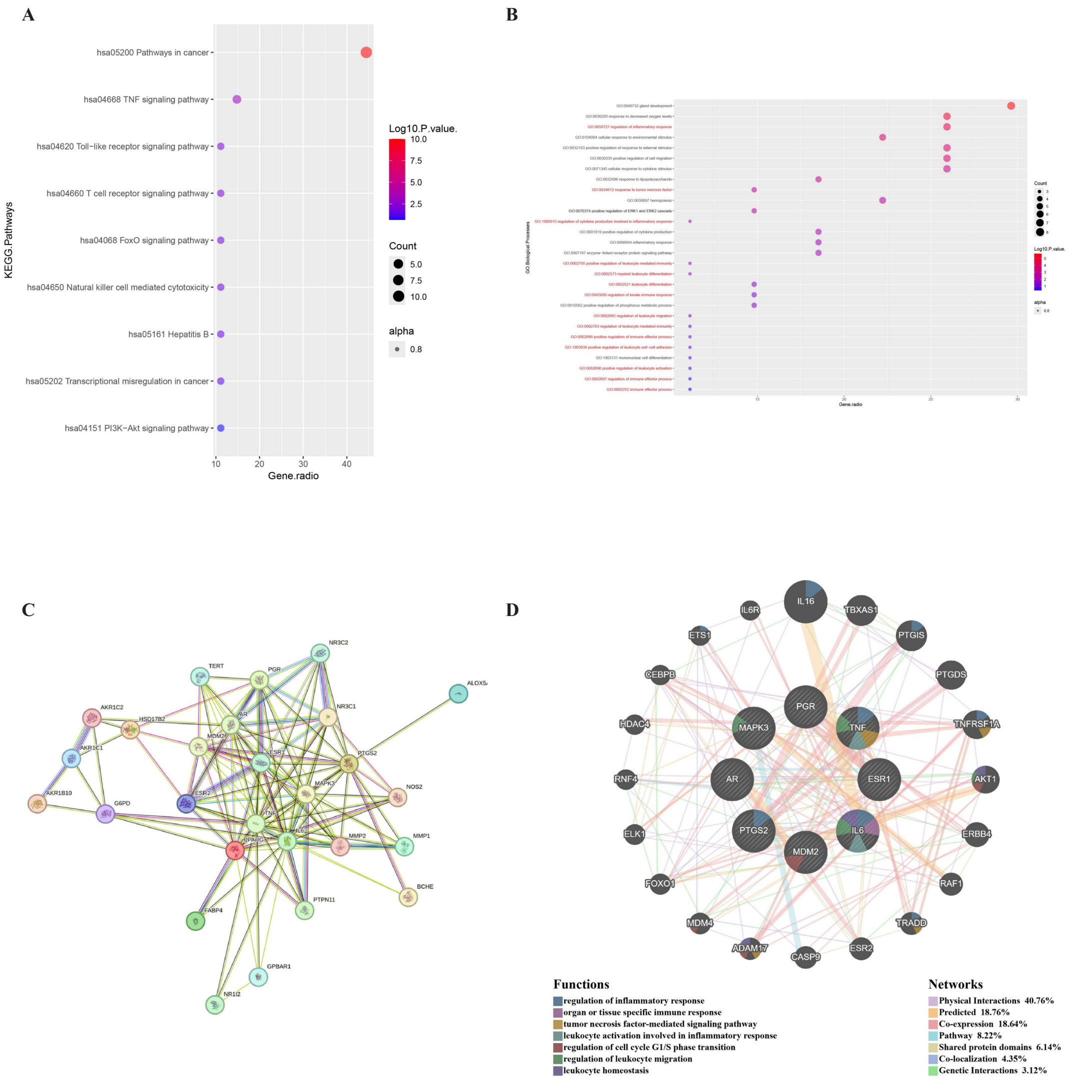

Part 2: Supplementary tables

Supplementary Table 1 Elution gradient table.

Time (min)	A (%)	B (%)
0	95	5
2.5	16	84
4	2	98
4.1	95	5
5.5	95	5

Supplementary Table 2 Mass spectrometry parameters information.

Parameters	Conditions
Curtain Gas (CUR)	30
Collision Gas (CAD)	9
IonSpray Voltage (IS)	-4500
Temperature (TEM)	450℃
Ion Source Gas 1 (GS1)	50
Ion Source Gas 2 (GS2)	55
Entrance Potential (EP)	-10
Collision Cell Exit Potential (CXP)	-12

Supplementary Table 3 Fragment information and quantification limits of each compound.

Compound	Q1 Mass (Da)	Q3 Mass (Da)	CE (volts)	DP (volts)	Ion mode
Liquiritin	417.2	255.0 (quantitation)	-26	-42	Negative
		135.1 (Identification)	-37	-42	
Glycyrrhetinic acid	469.4	425.4 (Quantitation)	-50	-60	Negative
		355.3 (Identification)	-64	-60	
Paeoniflorin	479.2	121.0 (quantitation)	-28	-80	Negative
		327.3 (Identification)	-19	-80	
Kansuinine A	729.3	121.1 (quantitation)	-17	-100	Negative
		669.3 (Identification)	-10	-100	
Glycyrrhizic acid	821.2	351.1 (quantitation)	-58	-50	Negative
		193.1 (Identification)	-59	-50	
Bendrofluazide (internal standard)	420.1	289.0	-33	-122	Negative
6-HF (internal standard)	237.0	193.0	-27	-98	Negative

Supplementary Table 4 Extraction recovery and matrix effect results of five components.

Compound	Concentration (ng/mL)	Extraction recovery		Matrix effect	
		Accuracy (%)	Precision (RSD, %)	Accuracy (%)	Precision (RSD, %)
Paeoniflorin	4.5	79.1	2.3	115.7	7.0
	30	89.1	2.3	100.4	6.1
	600	89.8	3.5	81.0	3.2
Liquiritin	0.15	99.4	0.9	69.2	5.4
	1	87.8	1.8	60.0	2.0
	20	86.4	2.0	60.4	2.4
Glycyrrhizic acid	1.5	87.1	2.9	108.9	4.3
	10	83.8	0.9	104.0	0.9
	200	81.9	1.7	100.3	0.7
Glycyrrhetinic acid	1.5	115.5	6.3	129.6	2.0
	10	88.8	5.1	153.5	5.3
	200	88.4	2.8	133.6	7.1
Kansuinine A	0.15	119.8	5.3	86.1	8.3
	1	104.9	7.1	95.5	2.4
	20	104.4	7.7	84.6	1.6

Supplementary Table 5 Regression equations and quantification limits for five components standard curves.

Compound	Standard Curve Equation	r	Linear Range (ng/mL)	LLOQ (ng/mL)
Paeoniflorin	y = 0.00202x + 0.000271	0.9960	1.5-750.0	1.5
Liquiritin	y = 0.17x + 0.0081	0.9959	0.05-25.0	0.05
Glycyrrhizic acid	y = 0.0491x + 0.00531	0.9951	0.5-250.0	0.5
Glycyrrhetinic acid	y = 0.027x + 0.00195	0.9963	0.5-250.0	0.5
Kansuinine A	y = 0.0107x - 0.000133	0.9960	0.05-25.0	0.05

Supplementary Table 6 Intra-day accuracy and precision experimental results for five components (n = 3).

Compound	Concentration (ng/mL)	Accuracy (%)	Precision (RSD, %)
	4.5	86.8	4.3
Paeoniflorin	30	100.8	1.0
	600	89.3	2.5
	0.15	88.7	2.6
Liquiritin	1	91.5	3.7
	20	98.7	3.1
	1.5	94.2	3.9
Glycyrrhizic acid	10	100.2	3.1
	200	105.7	2.6
	1.5	108.4	1.5
Glycyrrhetinic acid	10	112.3	1.9
	200	111.2	3.2
	0.15	86.4	6.2
Kansuinine A	1	92.9	3.3
	20	91.3	7.0

Supplementary Table 7 Stability of five components (n = 3).

Compound	Concentration (ng/mL)	Room temperature incubation for 2 hours		Repeated freeze-thaw cycles three times		Incubation in the autosampler for 6 hours	
		Accuracy (%)	Precision(RSD, %)	Accuracy (%)	Precision(RSD, %)	Accuracy (%)	Precision(RSD, %)
Paeoniflorin	4.5	95.5	3.8	91.8	2.5	91.3	8.1
	30	85.6	3.4	102.7	7.5	98.2	9.3
	600	86.6	4.5	85.9	6.2	90.3	1.0
Liquiritin	0.15	105.6	1.6	88.9	10.9	86.7	1.3
	1	86.3	4.4	89.5	5.8	94.8	1.3
	20	97.5	3.6	98.7	9.1	103.2	2.8
Glycyrrhizic acid	1.5	112.4	3.8	93.6	2.5	104.0	1.9
	10	94.6	3.9	97.0	6.6	106.0	5.7
	200	106.5	7.3	108.8	6.9	110.0	2.7
Glycyrrhetinic acid	1.5	98.7	12.4	94.7	2.5	98.9	13.3
	10	89.1	0.6	98.8	4.7	98.9	2.3
	200	104.2	5.6	114.7	3.6	99.2	2.8
Kansuinine A	0.15	63.4	1.3	70.9	9.0	69.5	5.5
	1	73.6	2.6	74.7	7.3	80.7	5.3
	20	80.7	5.0	73.7	7.1	81.8	2.5

Supplementary Table 8 The reverse transcription system.

Ingredients	Volume (μl)
Reverse transcriptase	1
RNA Enzyme Inhibitors	1
Oligo (DT)	1
Deoxynucleotide triphosphate	2
5× reverse transcriptase buffer	4
DEPC water	8
Total RNA	3

Supplementary Table 9 The amplification system.

Ingredients	ANP (μl)	BNP (μl)	NPR-A (μl)	PKGII (μl)
Upstream primers	0.3	0.5	0.5	0.4
Downstream primers	0.3	0.5	0.5	0.4
DEPC water	6.4	6.0	6.0	5.2
Fluorescent dye mix	10.0	10.0	10.0	10
cDNA	5.0	3.0	3.0	4
Temperature of annealing	60 °C	55 °C	55 °C	60 °C

Supplementary Table 10 Primer sequences.

Primer names	Sequence (5’-3’)
ANP	up 5’-ACCTGCTAGACCACCTGGAG-3’<br>down 5’-CTTCATCGGTCTGCTCGCTC-3’
BNP	up 5’-TCCAGGTGGTCTAGCAGGTTCTTG-3’<br>down 5’-GCCTTGGTCCTTTGAGAGCTGTC-3’
NPR-A	up 5’-ACAGCAGCAACATCCTGGACAAC-3’<br>down 5’-GGAGTGAGGCAGAATCTGGTAAAGC-3’
PKGII	up 5’-CTGGATGTTCACCGCAAGACCTC-3’<br>down 5’-TCCTTCCTGACCCTCGCTTTCTC-3’
β-actin	up 5’-GCAGTTGGTTGGAGCAA-3’<br>down 5’-ATGCCGTGGATACTTGGA-3’

Supplementary Table 11 Flow cytometry antibody information.

Antibody	Fluorochrome	Manufacturer and item number
CD3	AF-488	Biolegend, no. 201406
CD4	BV 786	BD, no. 740912
CD8	BUV 737	BD, no. 741771
CD161	BV 421	Biolegend, no. 744049
CD25	PE	BD, 554866
CD28	BV 711	BD, no.742586
Foxp3	APC	Ebioscience, no. 17-5773-82
CD45	BUV 395	BD, no. 740258
FVS 780	FVS 780	BD, no. 565388

Supplementary Table 12 Cell cycle antibody information.

Antibody	Fluorochrome	Manufacturer and item number
CD3	AF-488	Biolegend, no. 201406
CD4	BV 786	BD, no. 740912
CD8	BUV 737	BD, no. 741771
FVS 780	FVS 780	BD, no. 565388
Ki-67	Ki-67	Thermo, no.12-5698-82
7-AAD	7-AAD	Biolegend, no. 420403

Supplementary Table 13 The number of active components and potential drug therapeutic targets of GBD.

Herbs	Latin name	Number of active components	Number of potential targets
GS (Kansui)	*Euphorbia kansui* Liou ex S.B.Ho.	40	606
GC (Liquorice)	*Glycyrrhiza uralensis* Fisch.	142	875
BS (Radix paeoniae alba)	*Paeonia lactiflora* Pall.	50	541
BX (Pinellia ternata)	*Pinellia ternata* (Thunb.) Makino.	23	485
FM (honey)	*Apis cerana* Fabricius.	32	313

Supplementary Table 14 The top 10% active components of each herb.

Herbs	Components	Betweenness Centrality	Closeness Centrality	Degree	Chemical name
BS	BS21	0.020	0.367	112	Pyrethrin Ii
BS	BS43	0.022	0.368	111	paeonenoide A
BS	BS20	0.031	0.367	110	Pyrethrin I
BS	BS4	0.013	0.361	74	albiflorin_qt
BS	BS3	0.013	0.361	74	albiflorin
BX	BX21	0.025	0.362	111	Noopept
BX	BX1	0.034	0.365	110	(3S,6S)-3-(benzyl)-6-(4-hydroxybenzyl)piperazine-2,5-quinone
BX	BX6	0.029	0.364	107	Cavidine
FM	FM27	0.005	0.367	104	Apigenin
FM	FM25	0.004	0.366	104	Luteolin
FM	FM24	0.005	0.367	104	Morin
FM	FM20	0.005	0.367	104	Galangin
FM	FM19	0.006	0.368	104	Chrysin
GC	GC17	0.040	0.369	141	3,22-Dihydroxy-11-oxo-delta(12)-oleanene-27-alpha-methoxycarbonyl-29-oic acid
GC	GC81	0.014	0.368	114	shinpterocarpin
GC	GC30	0.014	0.370	114	euchrenone
GC	GC23	0.023	0.372	114	7-Acetoxy-2-methylisoflavo

					ne
GC	GC118	0.015	0.368	112	glycyrdione B
GC	GC38	0.021	0.371	112	Glabranin
GC	GC83	0.013	0.366	109	Vestitol
GC	GC45	0.009	0.366	109	Glyasperins M
GC	GC67	0.010	0.365	108	licoisoflavanone
GC	GC40	0.011	0.364	108	Glabridin
GC	GC77	0.030	0.363	106	Phaseolinisoflavan
GC	GC120	0.011	0.363	106	glycyrrhisoflvanone
GC	GC52	0.009	0.370	106	HMO
GC	GC46	0.017	0.364	106	Glycyrin
GC	GC19	0.012	0.365	106	3'-Methoxyglabridin
GS	GS26	0.038	0.370	141	Kansuiphorin D
GS	GS13	0.026	0.364	114	20-O-(Decanoyl)Ingenol
GS	GS11	0.012	0.364	112	20-O-(2'E,4â€™Z-Decadienoyl)Ingenol
GS	GS21	0.016	0.359	110	5-O-(2'E,4'E-Decadienoyl)Ingenol

Supplementary Table 15 The number of MA-related targets in databases.

Datasets	Number
Drugbank	35
GeneCard	547
OMIM	69
PharmGKB	135
TTD	1

Supplementary Table 16 The number of DEGs of MA in GEO datasets.

Datasets	Number
GSE73168	1041
GSE39204	81
GSE15831	22

Part 3: Prediction of the mechanism of glycyrrhetinic acid

G1. Material and methods

G1.1 Acquisition of key therapeutic targets of glycyrrhetinic acid

The MA-related targets screened in **2.2.3** and the DEGs of MA screened in **2.2.4** were merged, and then intersected with the therapeutic targets of glycyrrhetinic acid screened in **2.2.2** using the Venn online analysis tool (https://bioinformatics.psb.ugent.be/webtools/Venn/). The acquired intersection is the key therapeutic target of glycyrrhetinic acid in treating of MA.

G1.2 Gene Ontology Biological Processes (GO BP) and Kyoto Encyclopedia of Genes and Genomes (KEGG) enrichment analysis

The intersection genes obtained from **G1.1** were analysed for GO BP and KEGG enrichment using the Metascape (https://metascape.org, Metascape 3.5, updated on February 2024, accessed March 2025) (Zhou et al., 2019) online analysis tool. The results were visualized using the "ggplot2" package in the R language.

G1.2 Topological and cluster analyses of the protein-protein interaction (PPI) network

The key therapeutic targets of glycyrrhetinic acid obtained from **G1.1** were imported into the STRING online database (https://cn.string-db.org/, accessed March 2025) (Szklarczyk et al., 2023) to analyse PPI, and the default parameters were maintained. Then, the generated PPI network TSV file was loaded into Cytoscape 3.9.1 software to draw the PPI network diagram.

G1.3 Selection and analysis of hub genes

The hub genes were identified using the cytoHubba plug-in of Cytoscape. We used six common algorithms (Degree, MCC, MNC, EPC, Radiality, and Closeness) to evaluate and select the hub genes. Subsequently, we constructed a co-expression network of these hub genes via GeneMANIA (http://www.genemania.org/, accessed March 2025) (Warde-Farley et al., 2010), which is a reliable tool to identify internal associations in gene sets. GeneMANIA is an exceptionally large functional association database that finds other genes related to a set of input genes. Given a list of query genes, GeneMANIA finds functionally similar genes using extensive genomic and proteomic data. In this model, it weights each functional genomic dataset based on the predicted value of the query. Another use of GeneMANIA is gene function prediction. Given a query list of hub genes, GeneMANIA extends the list with functionally similar genes that it identifies using available genomics and proteomics data. Thus, through GeneMANIA, we predicted the functions of the hub genes.

G2. Results

G2.1 GO and KEGG analysis of intersecting targets

The 27 key therapeutic targets were obtained by intersection of disease-related targets with drug targets for glycyrrhetinic acid ***(Supplementary File 1 (Worksheet 14:***

GA-Target of intersection)).

The 27 intersection genes were imported into the Metascape database for enrichment analysis, and the filtering conditions were according to the default options. The detailed results of the GO and KEGG enrichment analyses are shown in ***Supplementary File 1 (Worksheet 15: GA-KEGG pathways; Worksheet 16: GA-GO Biological Processe)***. The results related to the research directions were visualised using the "ggplot2" package in R language, as shown in ***Supplementary Figure 9(A, B)***.

Compared with the KEGG enrichment results of glycyrrhetinic acid and GBD, we found that GBD basically covered the KEGG pathways enriched by glycyrrhetinic acid. However, the KEGG enrichment pathways of glycyrrhetinic acid were more inclined to inflammatory, immune and anti-tumor related pathways.

Comparing the GO BP enrichment results of glycyrrhetinic acid and GBD, we found that GBD also basically covered the GO BP enriched by glycyrrhetinic acid. However, in the glycyrrhetinic acid enrichment results, its anti-inflammatory and immune-related cell functions became more prominent (red marks).

G2.2 Selection and analysis of hub genes

The PPI network (***upplementary Figure 9C***) of 27 intersection genes obtained in STRING was imported into Cytoscape 3.10.1, and the hub genes were screened using the cytoHubba plug-in. We selected six algorithms (Degree, MCC, MNC, EPC, Radiality, and Closeness) to evaluate and screen hub genes, and each algorithm took the top 10. The screening details of the hub genes are shown in ***Supplementary File 1 (Worksheet 17: GA-Hub genes prediction)***. The Draw Venn Diagram (https://bioinformatics.psb.ugent.be/webtools/Venn/) was used to obtain 8 hub genes , which were IL6, TNF, ESR1, AR, MAPK3, MDM2, PTGS2 and PGR.

GeneMANIA was used to predict functionally similar genes of hub genes. We obtained 20 similar genes of hub genes ***(Supplementary Figure 9D)***. The hub genes were located in the inner circle, while the predicted genes were in the outer circle. In this network, the Physical Interactions, Predicted, Co-expression, Pathway, Shared protein domains, Co-localisation, Genetic Interactions values were 40.70%, 18.76%, 18.64%, 8.22%, 6.14%, 4.35% and 3.12% respectively. These genes are related to the regulation of immune and inflammatory response.
